# Parallel multilink group joint ICA (pmg-jICA): Fusion of 3D structural and 4D functional data across multiple resting fMRI networks

**DOI:** 10.1162/NETN.a.575

**Published:** 2026-08-25

**Authors:** K. M. Ibrahim Khalilullah, Souvik Phadikar, Oktay Agcaoglu, Jing Sui, Marlena Duda, Tülay Adali, Vince D. Calhoun

**Affiliations:** Tri-institutional Center for Translational Research in Neuroimaging and Data Science (TReNDS), Georgia State University, Georgia Institute of Technology, Emory University, Atlanta, GA, USA; Department of Electrical and Computer Engineering, University of Maryland, Baltimore County, Baltimore, MD, USA

**Keywords:** Multimodal fusion, Group ICA, Joint ICA, pmg-jICA, Gray matter, fMRI, Intrinsic connectivity network, Joint spatial maps and subject loadings

## Abstract

Integrating neuroimaging data enhances our understanding of the brain. Structural magnetic resonance imaging (sMRI) offers high-resolution anatomical detail, while functional MRI (fMRI) captures dynamic neural activity. Combining these modalities can reveal significant structure–function relationships in the brain. However, existing approaches typically link sMRI to only a single fMRI network, overlooking the spatial complexity of multiple networks and thereby missing distributed structure–function relationships. To address this limitation, we present parallel multilink group joint ICA (pmg-jICA), a data-driven framework that fuses gray matter images from sMRI with multiple intrinsic fMRI networks within a single model. pmg-jICA captures cross-network structure–function coupling, preserves subject-specific variability, and enables robust group-level statistical analyses. To demonstrate the approach, we applied pmg-jICA to an Alzheimer’s disease (AD) dataset, recovering linked structural and functional components for 53 brain networks. Notably, patients with AD exhibited alterations in subcortical, cognitive control and visual regions. Importantly, the subject loadings enabled the computation of functional network connectivity, revealing additional alterations in subcortical, visual, and cognitive control systems. Overall, our results demonstrate that pmg-jICA overcomes key limitations of existing multimodal fusion techniques, yielding deeper insights into structure–function disruptions in AD and potentially offering a flexible framework for studying other neurological and psychiatric disorders.

## Author Summary

We present parallel multilink group joint ICA (pmg-jICA), a multimodal fusion framework for jointly analyzing structural MRI (sMRI) and multiple functional MRI networks. Unlike conventional approaches that link structure to a single functional map, pmg-jICA captures network-specific and internetwork structure–function relationships by integrating gray matter with multiple intrinsic connectivity networks in a unified model. Applied to Alzheimer’s disease data, the method reveals distributed structural and functional alterations across subcortical, cognitive control, auditory, visual, and cerebellar systems. It preserves subject-level variability, enabling robust group comparisons and functional network connectivity analysis. Overall, pmg-jICA provides richer and more interpretable multimodal patterns than unimodal approaches and offers a flexible framework for studying complex brain disorders.

## INTRODUCTION

Structural magnetic resonance imaging (sMRI) measures the anatomy and morphology of brain structures, providing high-resolution detail, whereas functional magnetic resonance imaging (fMRI) captures the blood oxygenation level dependent (BOLD) signals to reveals the brain’s functional organization. The relationship between these two modalities, commonly referred to as structure–function coupling, reflects the extent to which anatomical connectivity or gray matter (GM) organization support and constrain patterns of functional activity and communication across brain networks. Recent work demonstrates clear benefits of integrating sMRI and fMRI for detecting disease-related alterations and improving diagnostic performance. For example, Park et al. reported that integrating cortical thickness with default mode fMRI connectivity improves diagnostic accuracy relative to unimodal analyses (Park et al., 2017). Reviews of multimodal fusion methods likewise emphasize that integrating sMRI and fMRI reveals coupled structural–functional mechanisms that are not recoverable from either modality alone (Calhoun & Sui, 2016; Sui et al., 2023). This prior evidence strongly motivates the development of joint models such as parallel multilink group joint independent component analysis (pmg-jICA), which aims to identify coherent cross-modal components that reflect shared structure–function alterations in Alzheimer’s disease (AD). Integrating sMRI with fMRI links the structural substrates with its functional information, revealing mechanisms that neither modality alone can capture. However, multimodal fusion is challenging because it requires integrating data that differ in scale, dimensionality, and statistical properties.

The current landscape of multimodal neuroimaging research has seen significant progress, yet challenges remain in seamlessly integrating information from multimodal data. Luo et al. demonstrated a structural–functional relationship using a dataset over 1,500 individuals, employing a two-step process with separate analyses rather than a joint approach (Luo et al., 2020). Similarly, Sendi et al. investigated the transition from a normal cognition to very mild AD using separate multimodal analyses (Sendi et al., 2020). The joint analysis of multiple imaging data types from the same individual has been shown to be particularly valuable in this context. Qi et al. developed a three-way parallel group independent component analysis (ICA) fusion method to distinguish between schizophrenia and control subjects while linking data with different dimensionalities (Qi et al., 2022). Additionally, Duan et al. introduced a multimodal fusion approach known as aNy-way ICA, capable of detecting interconnected sources across any number of modalities without imposing orthogonality constraints on the sources (Duan et al., 2020).

Overall, many multimodal approaches often assume that different modalities originate from a shared distribution. In practice, high-dimensional fMRI data are frequently reduced to a single summary map for each subject—such as the amplitude of low-frequency fluctuations (ALFFs; Hare et al., 2017; Jia et al., 2021; Turner et al., 2012) or a representative intrinsic network like the default mode network. Using a highly summarized measure like the ALFFs can be lossy and may not capture the full spatial complexity of the fMRI data. This reduction can lead to a loss of detailed spatial information, making it challenging to integrate with other modalities that might benefit from more nuanced spatial representations. An additional complication is that datasets from the same individual are not statistically independent or identically distributed, making direct integration even more difficult.

ICA has shown great promise in the analysis of fMRI data and is widely used. When applied to fMRI, ICA can effectively segregate sources that are independent spatially or temporally, performing well under appropriate assumptions (Adali et al., 2014; Calhoun et al., 2001, 2021; Calhoun & de Lacy, 2017). Extensions of ICA, such as joint ICA and parallel ICA, are widely used for multimodal fusion of fMRI, sMRI, and electroencephalography (EEG), enabling joint analysis of multiple modalities. These approaches enhance our understanding of the human brain and its disorders, offering a more comprehensive perspective than studies focusing on a single modality (Calhoun & Sui, 2016; Eichele et al., 2008; Phadikar et al., 2025; Sendi et al., 2020).

Our previous model, parallel multilink joint ICA (pml-jICA), provides a powerful approach for fusing GM maps with multiple functional networks simultaneously while also allowing for different distributions (Khalilullah et al., 2023). However, although pml-jICA incorporates multiple brain networks, it does not fully capture internetwork interactions, nor does it optimize the mapping between individual sets of linked functional networks and their associated structural patterns. This optimization is important because different functional networks may relate to distinct structural substrates across individuals. If the fusion method does not account for such network-specific variability, it may blur important structure–function relationships and reduce sensitivity to subtle but clinically meaningful alterations.

To address this gap, we extend ICA as a unifying framework for multimodal fusion and introduce a method that combines concepts from group ICA and pml-jICA. The new approach, pmg-jICA, enables fusion of multiple functional brain networks and their temporal information with structural information (GM images from sMRI). By tailoring the fusion to individual network–structure pairings, pmg-jICA preserves network-specific structure–function relationships and prevents them from being averaged out across unrelated networks. This results in a more precise characterization of how structural degeneration relates to functional disruption.

We applied this approach to study a dataset collected from individuals with AD (LaMontagne et al., 2019). AD is one of the most common and devastating neurodegenerative diseases, affecting millions of people worldwide. It is characterized by progressive cognitive decline and significant structural and functional brain changes. Studying AD presents a critical challenge for neuroscience due to the complex interaction between structural degeneration and functional impairments. The proposed pmg-jICA estimates joint components that help explain these complexities.

The proposed framework enables *network-specific* fusion with GM, estimation of functional network connectivity (FNC), and *network-specific* structural decompositions. A key strength of the proposed method is its ability to obtain subject-specific spatial maps via back-reconstruction from the fused structure–function model. These subject-level maps can then be examined for group differences or associations with clinical and behavioral variables. We demonstrate the utility of pmg-jICA by applying it to an AD dataset, recovering linked structure–function outputs across 53 brain networks identified with the NeuroMark spatially constrained ICA pipeline (Du et al., 2020).

## METHODS

### pml-jICA

The pml-jICA framework (Khalilullah et al., 2023) was developed to jointly decompose structural and functional brain imaging data by linking a set of fMRI networks with GM images into a common set of independent components. In this method, data from each modality are first reduced via principal component analysis (PCA) and then analyzed together using Infomax ICA, which estimates a single mixing matrix shared across both modalities and subjects. It assumes a fixed, global linear relationship between fMRI and sMRI across subjects. Thus, once the joint components are estimated, all subjects share a common mixing matrix between fMRI and sMRI. Hence, pml-jICA cannot explicitly capture (a) internetwork interactions and (b) subject-specific variations in how fMRI links to sMRI. For details, readers may refer to the original manuscript (Khalilullah et al., 2023).

### pmg-jICA

**Figure 1.**
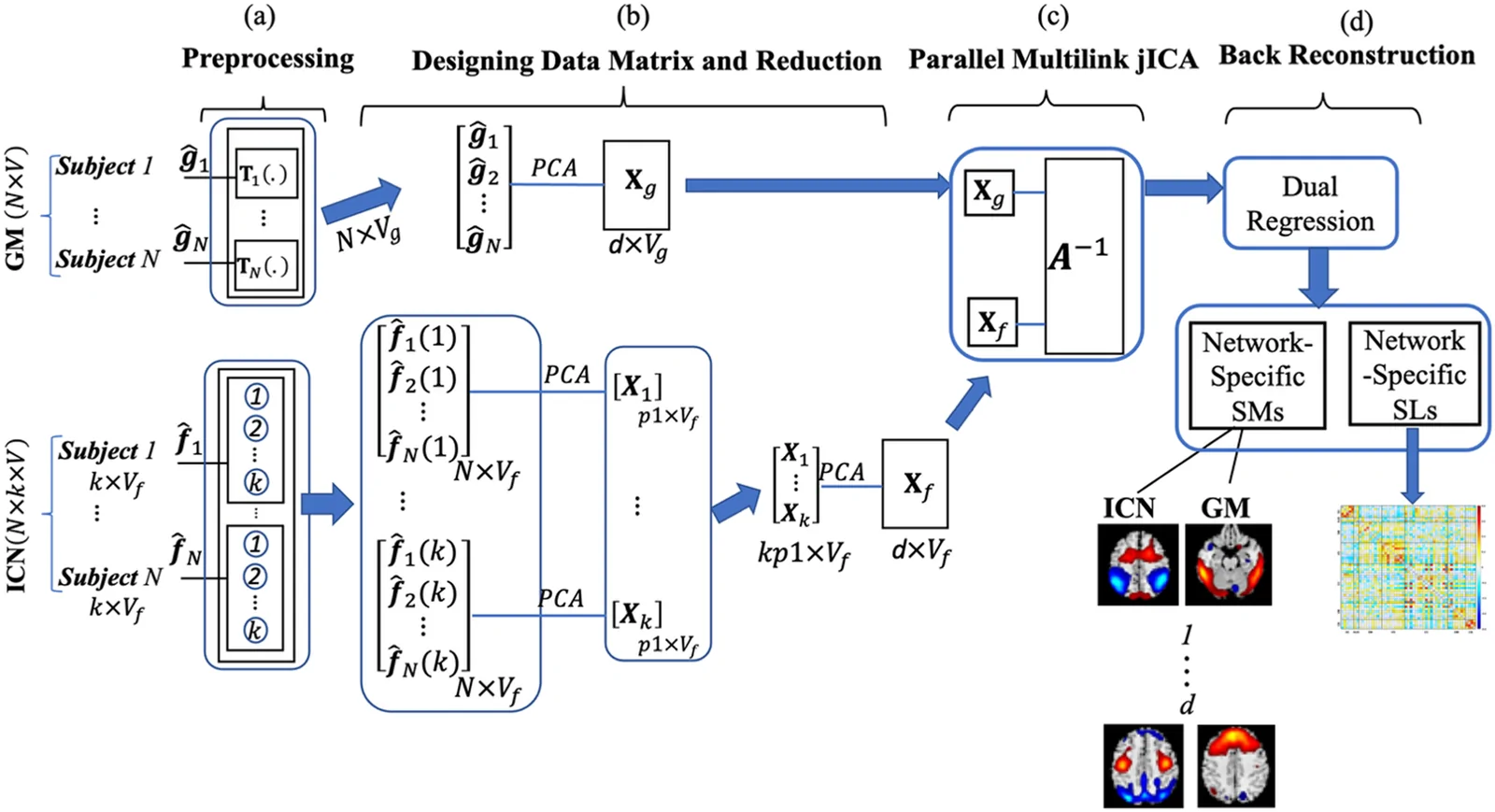
Pipeline of the pmg-jICA framework. The pipeline illustrates the workflow of the pmg-jICA method for multimodal integration of GM and ICNs. The process begins with preprocessing, where GM and fMRI images undergo spatial normalization and smoothing. Subject-specific ICNs are then generated using spatially constrained ICA applied to fMRI images with NeuroMark 1.0 templates. Next, we pick one ICN from each subject at a time and a data matrix is created separately for all ICNs. PCA is applied to each data matrix to merge subject information. The reduced data matrices are concatenated, and a second-level PCA is applied to reduce the within-network redundancy. Similarly, a single PCA is performed on stacked GM images from all subjects. Finally, we applied pml-jICA to reduced data matrices from GM and ICNs and estimated joint independent components. Next, dual regression is applied to the joint components generating modality-specific spatial maps as well as subject loadings.

To address these limitations, we extended pml-jICA into pmg-jICA. The framework introduces a network-specific multilink mapping step combined with a group ICA model. Unlike pml-jICA, which fuses one fMRI map with sMRI at a time, pmg-jICA fuses across all intrinsic connectivity networks (ICNs) individually, allowing both between- and within-network relationships to be modeled. Instead of a single fMRI network, pmg-jICA uses multiple ICNs, which represent spatially distributed sets of brain regions that exhibit coherent resting-state activity and are commonly used to describe core functional systems (e.g., default mode, visual [VS]). The pmg-jICA pipeline is presented in Figure 1. The pipeline consists of four main stages, including (a) preprocessing, (b) designing data matrix—this design allows multiple ICNs to interact, (c) joint decomposition through pml-jICA—this step estimates joint independent components, and (d) back reconstruction through dual regression—this step computes subject-specific structural–functional component pairs optimized for each ICN.

#### Preprocessing.

This step performs basic preprocessing using a transformation **T**(.) of the raw data, primarily including spatial normalization, reslicing (to ensure all images have the same voxel resolution and orientation), and smoothing (to improve signal-to-noise ratio). After preprocessing, we applied spatially constrained ICA to the preprocessed fMRI data, where spatial priors were established using the Neuromark_fMRI_1.0 templates (Du et al., 2020), and 53 ICNs were generated for each subject. We used NeuroMark templates to ensure that the functional maps align with well-known, reliable networks, facilitating clinically meaningful interpretation of structure–function relationships.

#### Designing data matrix.

This step consists of designing the data matrix and performing dimensionality reduction. We designed the data matrix to incorporate all ICNs from all subjects, enabling group-level interactions and allowing joint fusion with GM data. For the fMRI modality, spatial maps from all subjects were organized into 53 matrices—one per NeuroMark ICN. For each ICN *k*, spatial maps from all subjects were organized into a single data matrix:

$$F_{k}=\left[\begin{array}{c} {\hat{f}}_{\left(1,k\right)}\\ {\hat{f}}_{\left(2,k\right)}\\ \vdots \\ {\hat{f}}_{\left(N,k\right)} \end{array}\right]$$ (1)

where *k* = [1, 2, …, *k*] represents the number of ICNs.

Next, each ICN-specific data matrix ***F***_*k*_ was reduced using *PCA* to capture network-specific variance across subjects:

$$X_{k}=PCA\left(F_{k}\right)$$ (2)

Here, ***X***_*k*_ is the reduced matrix of size of *p*1 × *V*_*f*_, where, *p*1 is the top principal components (PCs) retained based on cumulative variance (typically 90%–95%; Du et al., 2020; Li et al., 2007) and *V*_*f*_ is the number of voxels of ICN *k*. In this study, the same number of PCs, *p*1, was used for all ICN-specific data matrix ***X***_*k*_. This step compresses subject information and reduces within-network redundancy while preserving the subject-level details. Prior to PCA, we *z*-scored each voxel across subjects—subtracting its mean and dividing by its standard deviation. This step standardizes the voxel contributions but does not treat voxels as statistically independent. Spatial dependence among neighboring voxels remains intact because *z*-scoring was performed independently at each voxel across subjects and does not alter within-map spatial covariance.

Next, we concatenated the reduced data matrices (***X***_*k*_) for all 53 ICNs, and a second-level PCA was applied to concatenated data matrix to further condense it to the target number of components, *d*. The *d* × *V*_*f*_ reduced matrix ***X***_*f*_ can be expressed as follows:

$$X_{f}=PCA\left(\left[\begin{array}{c} X_{1}\\ \vdots \\ X_{k} \end{array}\right]\right)$$ (3)

We employed two levels of PCA to reduce the computational load of directly taking all subject’s data and identify common spatial patterns across a group of individual ICNs for all subjects. This two-stage PCA is standard in group ICA and multimodal fusion, ensuring computational efficiency while preserving the dominant variance structure across both subjects and networks (Qi et al., 2019; Sui et al., 2012).

Similarly, a single *PCA* was performed on stacked GM images from all subjects:

$$X_{g}=PCA\left(\left[\begin{array}{c} {\hat{g}}_{1}\\ {\hat{g}}_{2}\\ \vdots \\ {\hat{g}}_{N} \end{array}\right]\right)$$ (4)

where ***X***_*g*_ is the *d* × *V*_*g*_ reduced data matrix that aligns with fMRI data matrix ***X***_*f*_. Where *V*_*g*_ refers to the number of voxels in GM images. Both ***X***_*f*_ and ***X***_*g*_ share a consistent voxel space to ensure spatial correspondence during fusion. Because GM and ICNs were reduced to PCA components before fusion, there is no voxel-wise concatenation between GM and fMRI, and therefore no requirement for identical voxel dimensions across modalities.

#### Joint decomposition using pml-jICA.

In this stage, we jointly decomposed the ***X***_*f*_ and ***X***_*g*_ and estimated joint independent components. Because GM and ICN data follow different distributions, we used our previous algorithm pml-jICA, which performs alternating initialization and estimation, thereby relaxing the original jICA assumption of the same distribution across modalities. This also helps prevent one modality from dominating the other during estimation of the maximally independent components. Pml-jICA estimates mixing matrices ***A***_*g*_> and ***A***_*f*_ independently for the two modalities while enforcing a shared latent component structure across modalities through a joint optimization procedure. This design allows heterogeneous modalities to be modeled appropriately and reduces the risk that one modality dominates the joint decomposition.

In pml-jICA, ***X***_*g*_ and ***X***_*f*_ are initially expressed as follows:

$$X_{g}=A_{g}S_{g}\;\mathrm{and}\;X_{f}=A_{f}S_{f}$$ (5)

where ***A***_*g*_ and ***A***_*f*_ are mixing matrices, and ***S***_*g*_ and ***S***_*f*_ are the independent source maps of the GM and ICN, respectively. Pml-jICA estimates a shared mixing structure by alternating between modality-specific ICA updates and cross-modality alignment steps. At each iteration *t*

⇒  Given ***A***^(*t*)^, update $A_{g}^{\left(t+1\right)}$ and $A_{f}^{\left(t+1\right)}$ by maximizing the non-Gaussianity of *S*_*m*_ = (*A*^(*t*)^)^+^*X*_*m*_, *m* ∈ {*g*, *f*} using the Infomax natural gradient rule.
⇒  Compute variance-based weights *w*_*g*_ and *w*_*f*_ reflecting the relative signal power of each modality. The weights are defined as the total variance of the estimated source maps for each modality: *w*_*m*_ = *trace*(*cov*(*S*_*m*_)), *m* ∈ {*g*, *f*}.
⇒  Merge modality-specific updates to form a weighted joint matrix.
  $$A^{\left(t+1\right)}=\frac{w_{g}A_{g}^{\left(t+1\right)}+w_{f}A_{f}^{\left(t+1\right)}}{w_{g}+w_{f}}$$ (6)
⇒  Repeat until convergence (∥***A***^(*t*+1)^ − ***A***^(*t*)^∥/∥***A***^(*t*)^∥ < 10^−6^).

After convergence, the joint source maps (***S***) are obtained as follows:

$$S=A^{+}\left[X_{g}X_{f}\right]$$ (7)

where the “+” denotes the Moore–Penrose pseudoinverse.

#### Back-reconstruction through dual regression.

pml-jICA estimates a set of group-level joint source maps (***S***), which represent multimodal components jointly linking GM and ICNs. These joint components provide a common component basis shared across all networks. To obtain network-specific components, we applied a dual-regression-based back-reconstruction procedure (Erhardt et al., 2011) separately for each ICN. Specifically, for each ICN *k*, we reconstructed network-specific joint components ***S***_*k*_ by projecting the group-level joint components ***S*** onto the ICN-specific data matrix ${\hat{X}}_{k}$ (where ${\hat{X}}_{k}$ = [***X***_*g*_***X***_*f*_]_*k*_). In this formulation, the reconstructed ***S***_*k*_ were expressed as linear combinations of the group-level joint source maps. All ICNs therefore share the same group-level joint component basis, but each ICN has its own reconstructed spatial maps obtained through a network-specific projection.

The back-reconstruction was performed in two sequential regression steps. First, the network-specific subject loading matrix ***L***_*k*_ was estimated by projecting the ICN-specific data onto the group-level joint source maps:

$$L_{k}={\left[X_{g}X_{f}\right]}_{k}S^{+}$$ (8)

Each row of ***L***_*k*_ represents the expression of the joint multimodal components for an individual subject within ICN *k*. Second, the corresponding network-specific spatial maps (***S***_*k*_) were reconstructed by regressing the ICN-specific data (***X***_*k*_) on the estimated subject loadings:

$${\left[X_{g}X_{f}\right]}_{k}=L_{k}S_{k}+\varepsilon_{k}$$ (9)

where *ε*_*k*_ represents residual noise. ***S***_*k*_ contains modality-specific parts, namely, GM spatial map ***S***_*k*_^(g)^ and fMRI spatial map ***S***_*k*_^(f)^, corresponding to the structural and functional components of the joint sources. Detailed explanations of the regression and its derivations are provided in the Supporting Information. The pmg-jICA model and example code are available as part of the FIT toolbox for multimodal data fusion (https://github.com/trendscenter/fit).

The design of the data matrix in pmg-jICA allows multiple ICNs to interact, and reconstruction by regression analyses enables computation of subject-specific structural–functional relationship optimized for each ICN. In short, pml-jICA treats all ICNs as a single, homogenous feature space—effectively averaging distinct functional subsystems into one joint model. Pmg-jICA avoids this by modeling parallel links between sMRI and each ICN, followed by a group ICA that integrates these multiple mappings. This model captures network-specific structure–functional coupling that pml-jICA cannot recover. Particularly, pmg-jICA is necessary because it combines the strength of pml-jICA and group ICA to reveal subject-specific internetwork structure–function couplings that are blurred in a prior jICA model.

### Interpretation Pipeline

This step is not part of the main pmg-jICA pipeline but illustrates how the results can be interpreted. The pmg-jICA framework provides three primary outputs: network-specific subject loadings (***L***_*k*_, size: K × N × d), GM components ($S_{k}^{\left(g\right)}$, size: K × d × V), and fMRI components ($S_{k}^{\left(f\right)}$, size: K × d × V). Subject loadings are time series, whereas GM and fMRI components are expressed as spatial maps in this study. The subject loading for the *k*^th^ ICN is computed during the dual-regression stage as follows:

$$L_{k}=X_{k}S^{+}$$ (10)

Using this network-specific subject loadings, the GM and fMRI spatial maps for the ICN *k* are reconstructed separately for each modality,

$$S_{k}^{\left(g\right)}=L_{k}^{+}X_{g}$$ (11)

and

$$S_{k}^{\left(f\right)}=L_{k}^{+}X_{f}$$ (12)

where $S_{k}^{\left(g\right)}$ and $S_{k}^{\left(f\right)}$ denote the GM and fMRI spatial maps associated with ICN *k*, respectively.

**Figure 2.**
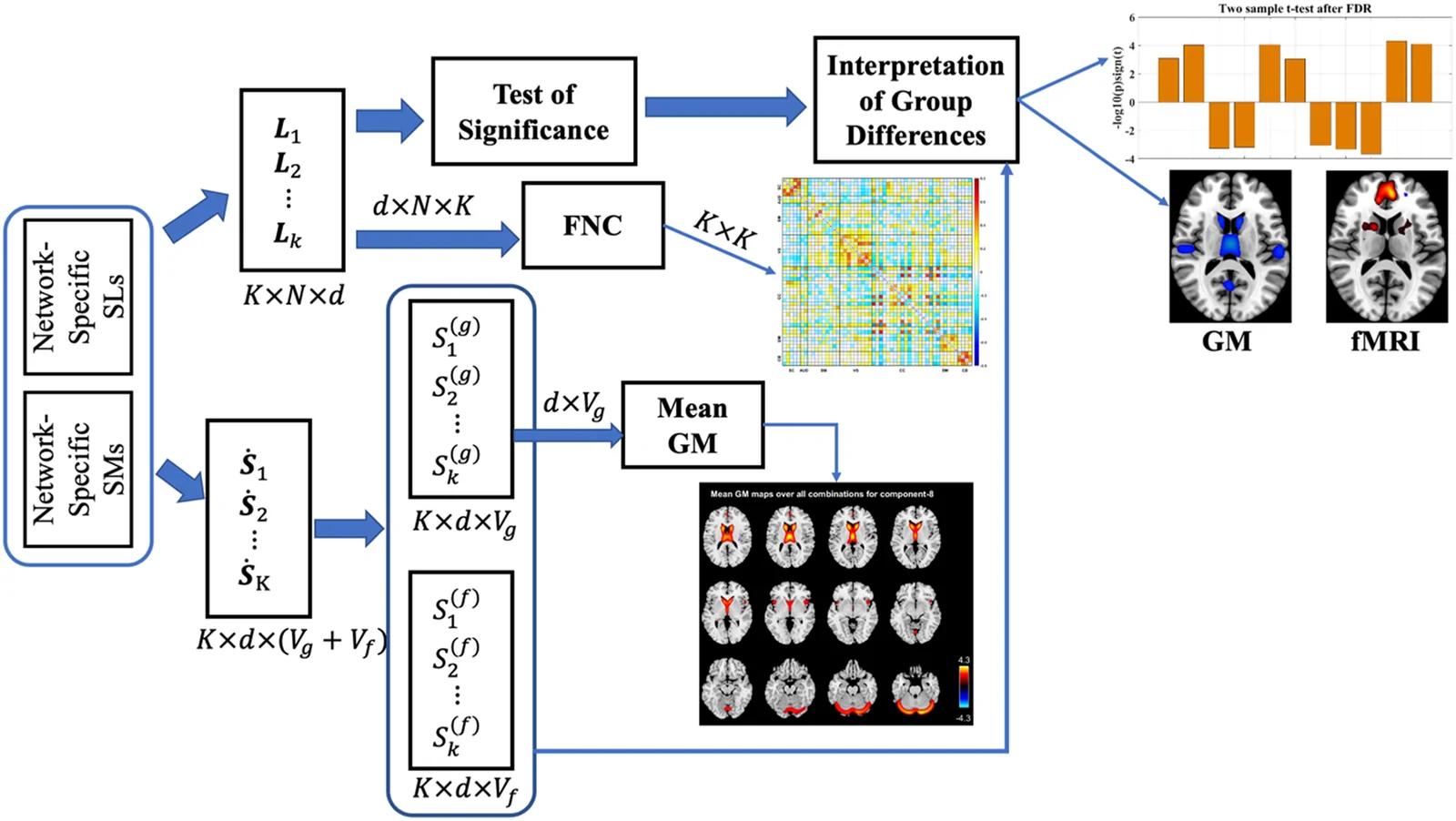
The figure shows the interpretation pipeline of the pmg-jICA results. The method produces GM components, fMRI components, and subject loadings for each ICN. The resulting GM component spatial maps represent *z*-scored structural component maps derived from T1-weighted MRI, and fMRI component spatial maps represent functional ICN spatial patterns derived from BOLD data. For each ICN, *k*, a two-sample *t* test was applied on subject loadings (***L***_*k*_) to assess the group differences between AD and HC groups. The corresponding GM–fMRI spatial maps of significant ***L***_*k*_ were then displayed to show the peak component expression. In the GM and fMRI components, spatial expression reflects the direction and strength of contribution of a region to a given component. A higher or lower expression indicates stronger or weaker involvement of that spatial pattern, respectively. When group comparisons are shown, warm colors (red/yellow) indicate regions with higher spatial expression in the AD group compared with those in the HC group, whereas cool colors (blue/cyan) indicate lower spatial expression in the AD group relative to that in the HC group. In addition, joint component-specific FNC matrices were computed and paired with the corresponding mean GM maps to reveal the functional connectivity patterns alongside their co-occurring structural regions.

Subject loadings represent component expression, which refers to how strongly a given brain pattern is present in an individual subject. A higher loading indicates that the subject shows the component pattern more strongly, whereas a lower loading indicates weaker expression of that pattern. In pmg-jICA, spatial maps of a GM component represent ICA-based spatial patterns of covarying GM intensity across subjects, whereas spatial maps of fMRI components represent ICN-based spatial patterns derived from BOLD data. Within the spatial maps, positive and negative voxel weights indicate the direction of contribution of each region to the component, reflecting how that region covaries with other regions across subjects. These signs are relative to the component. The analysis of these components is summarized in Figure 2.

In the Network-Specific Reconstructed Maps section, we first performed a two-sample *t* test on subject loadings of each of 53 ICN-GM pairs (*K* = 53 in our case) to identify network-specific alterations between healthy control (HC) and AD groups. The false discovery rate (FDR) approach was used to correct for multiple comparisons (*p* < 0.05). This analysis identifies the GM–fMRI component pairs that show significant group differences between AD and HC groups. Furthermore, for each significant component, we then examined the subject loadings in the two groups. To summarize the overall strength of each significant multimodal component within a group, we computed the sum of the subject loadings for that group. We compared these values between AD and HC groups to determine whether the corresponding multimodal pattern was stronger or weaker in the AD group relative to the HC group.

In the FNC section, we extended this analysis to examine how FNC relates to structural organization across components. For each component, *d*, we obtained a subject-loading matrix (***L***_*k*_) of size 53 × 260 (where *N* = 260 is the number of subjects). Component-specific (*d* = 10) FNC matrices were computed by applying Pearson correlations to each pair of ***L***_*k*_. Because the FNC represents internetwork connectivity at the whole-brain level, the corresponding GM spatial maps from all 53 ICNs were aggregated into single spatial map to match the global scope of the FNC representation. Finally, each FNC matrix was paired with its corresponding aggregated GM map, forming 10 FNC–GM pairs that capture shared structural contributions underlying global functional connectivity. These multimodal pairings reveal how variations in GM organization jointly relate to large-scale network interactions across subjects. This analysis was not performed to test group differences. Instead, it is intended to illustrate how the multimodal components identified by pmg-jICA link variations in GM organization with large-scale network interactions across subjects.

### Classification of the AD Group From the HC Group

We trained and tested classification models using 10-fold cross validation and out-of-field (OOF) framework. Multimodal features were derived from the pmg-jICA decomposition, including subject-specific GM loadings and subject-specific FNC matrices computed from ICN loadings. ICN and GM loadings were *z*-scored prior to feature construction. For each subject, selected FNC features were concatenated with GM loadings to form the final multimodal feature vector. FNC features were computed for each subject as pairwise correlations between ICN time courses, and a subset of informative FNC edges was selected using a training-only *t*-score criterion to identify connections showing the strongest group differences. Two classifiers: bagged decision tree ensembles (BagTree) and K-nearest neighbors (KNN) were evaluated, as these outperformed other tested classifiers in preliminary analyses. In each fold, 90% of the subjects were used for training and 10% were held out for testing. The hyperparameters were optimized using an inner fivefold cross-validation loop on the training data only. For the KNN classifier, the number of neighbors was tuned over the candidate set *K* = {1, 3, 5, 7, 9, 11, 15, 21}, with the optimal value selected based on maximum cross-validated training accuracy. The BagTree classifier parameters (number of trees = 600, minimum leaf size = 10, and maximum number of splits = 150) were fixed a priori and not tuned within the inner loop. Each subject contributed a single OOF prediction, and final performance metrics were computed from pooled OOF predictions. In addition, unimodal models using GM-only or FNC-only features were evaluated and compared with the multimodal models to assess the added value of integrating structural and functional information.

Classification performance was evaluated using the area under the receiver operating characteristic curve (AUC), accuracy, F1 score, and Brier score. The receiver operating characteristic (ROC) curve plots the true positive rate (sensitivity) against the false positive rate (1 − specificity) across varying decision thresholds, providing a threshold-independent assessment of classifier performance. The AUC summarizes the ROC curve into a single metric ranging from 0 to 1, where values closer to 1 indicate better discrimination between classes and 0.5 corresponds to chance-level performance. The F1 score, ranging from 0 to 1, represents the harmonic mean of precision and recall, with values closer to 1 indicating better balance between sensitivity and precision. The Brier score measures the mean-squared difference between predicted probabilities and true class labels, with lower values indicating better probabilistic calibration and overall predictive accuracy.

### Data and Preprocessing

This paper utilizes experimental data derived from the longitudinal OASIS-3 (LaMontagne et al., 2019). The dataset comprises MRI and positron emission tomography (PET) imaging data, along with relevant clinical data of 1,098 participants. Participants were instructed to remain quietly with open eyes while two 6-min resting-state BOLD sequences were recorded. The dataset includes a total of 2,165 MR sessions for sMRI with T1w scan type as well as 1,691 MR sessions with BOLD-resting-state scan type. Our analysis focused on utilizing sMRI and resting-state BOLD sequences.

We employed imaging data, demographic information, and clinical dementia rating (CDR) scores for each participant. For quality control, GM images were randomly grouped, and scans were flagged if the spatial correlation with the group average images fell below 0.95. This quality control process refined the dataset, reducing the initial 1,098 subjects to 784, comprising 648 HCs and 136 individuals with AD. We conducted an analysis on a conclusive subset, randomly choosing 260 participants from both the HC and AD groups within the refined dataset. This selection ensured equal sample sizes for both the control and patient cohorts. HCs were defined as cognitively normal with a CDR score of 0, while AD subjects were categorized as having AD dementia with a CDR ≥ 0.5.

The fMRI data underwent preprocessing in SPM12. Initially, motion correction followed by slice-timing correction was performed to address subject head motion and timing disparities in slice acquisition. These resliced images were further subjected to smoothing using a Gaussian kernel with a full width at half maximum (FWHM) of 6 mm. Simultaneously, sMRI data underwent segmentation and spatial normalization using the SPM unified segmentation approach, followed by Gaussian smoothing with an FWHM of 6 mm. The GM images analyzed in this study were derived from voxel-wise T1-weighted sMRI intensity values. Both the fMRI and sMRI data were spatially normalized to the Montreal Neurological Institute (MNI) standards and resliced into 3 mm × 3 mm × 3 mm isotropic voxels. This alignment ensures that corresponding voxels represent identical anatomical locations across modalities, which is essential for voxel-wise correspondence during joint analysis. Both the imaging data were normalized to unit variance across all subjects within each modality. The demographic and clinical details of the refined experimental data are succinctly presented in the Supporting Information.

## RESULTS

We applied the proposed pmg-jICA approach to the GMs and ICNs of 260 subjects and estimated 10 independent joint components for each of 53 ICNs. The number of estimated components was selected based on the empirical results using an information theoretic approach (Li et al., 2007). Specifically, applying the minimum description length criterion within the ICA framework, we found that beyond 10 components, an additional component primarily captured noise rather than meaningful brain signal.

### Network-Specific Reconstructed Maps

**Figure 3.**
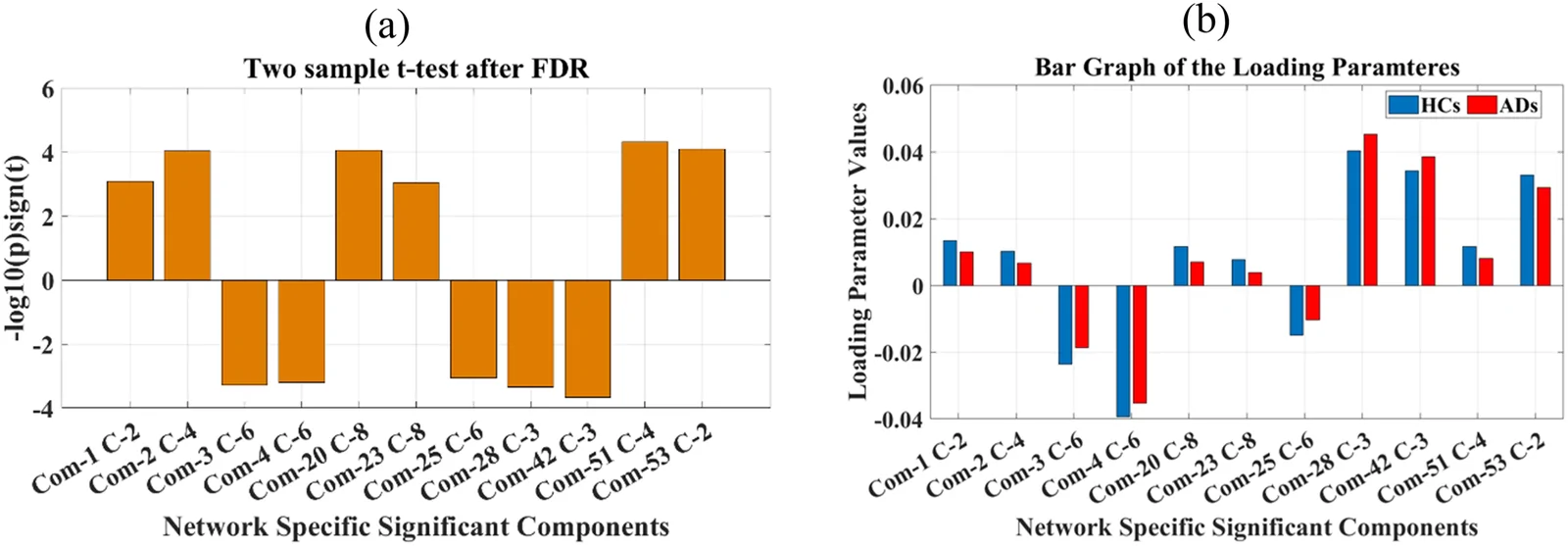
(A) Network-specific significant components are presented. Eleven combinations (out of 53 combinations, denoted as “Com-1,” etc.) are found as significantly distinct between the HC and AD groups (corrected at (*p* < 0.001). Furthermore, the significant combinations provide their significant components (denoted as “C-2,” etc.), which make the HC group distinct from the AD group. (B) Average subject loading parameter values of the significant components showing strength of loading parameters of the HC versus AD groups. Com-1, 2, 3, 4, 20, 23, 25, 51, and 53 show increased values in the HC group compared with the AD group, while Com-28 and 42 show a decrease in the HC group.

The collection of 53 reconstructed joint maps is referred to as “53 combinations” (com-1 to com-53). Each combination corresponds to one ICN. For each ICN, the pmg-jICA model estimates 10 joint independent components, each representing a distinct GM–fMRI pair. Therefore, each combination (Com-1 to Com-53) contains 10 GM–fMRI component pairs—one GM spatial map and one fMRI map per component—that jointly capture structure–function coupling specific to that ICN. Figure 3A presents the FDR-corrected two-sample *t*-test results, showing that 11 of the 53 combinations are significant (*p* < 0.001). Within each of these 11 combinations, we further identified the significant GM–fMRI component pairs, denoted as C-1 to C-10. Among them, nine combinations (com: 1, 2, 3, 4, 20, 23, 25, 51, 53) show reduction in absolute subject-loading parameters in the AD group compared with those of the HC group while other combinations (com: 28 and 42) show increased loadings in the AD group compared with those of the HC group, shown in Figure 3B. We note that alternative energy-based summaries such as squared loadings normalized by component variance may provide a complementary quantitative formulation and will be explored in future work.

**Figure 4.**
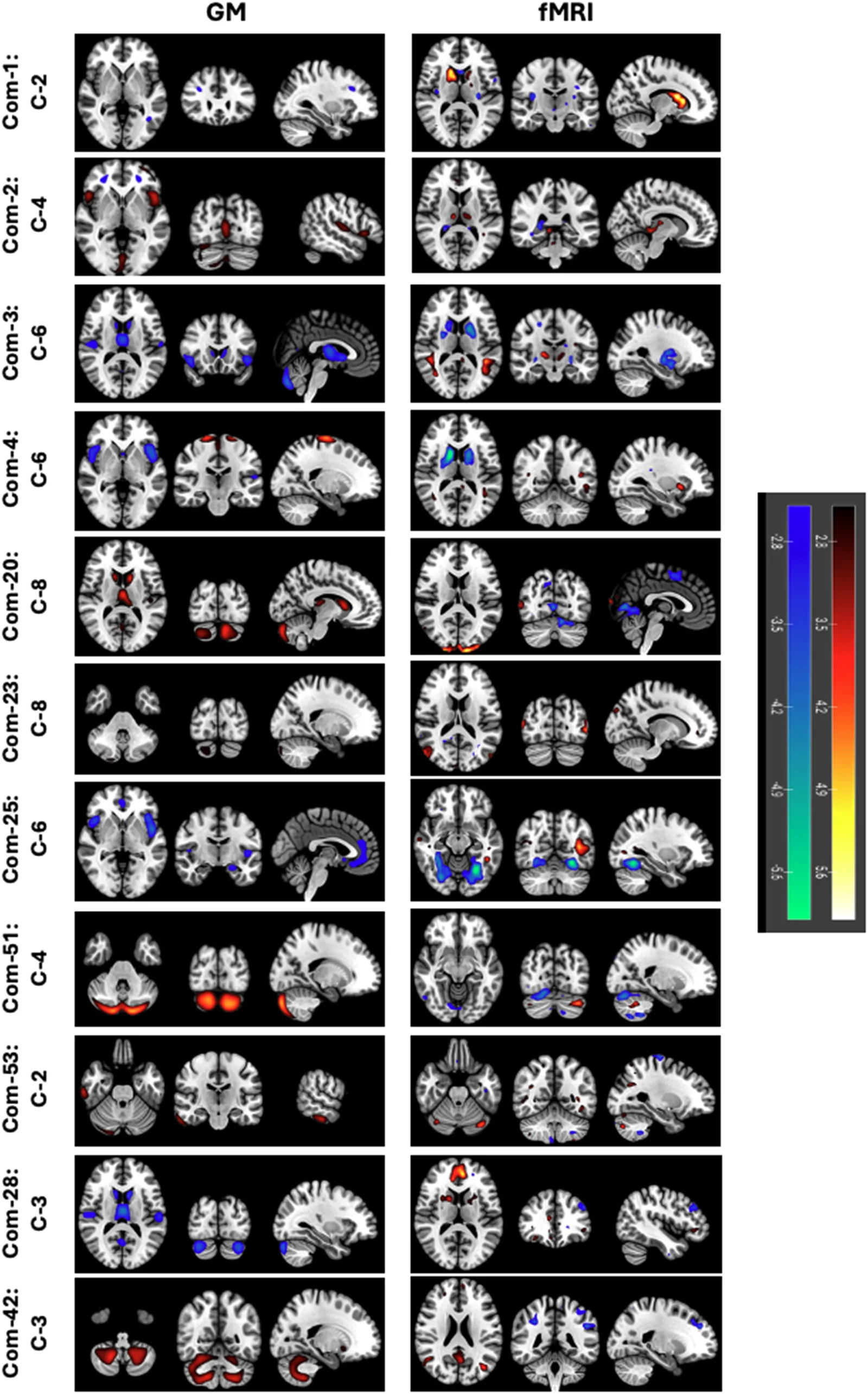
This figure shows the joint spatial maps of 11 significant GM–fMRI component pairs identified through a two-sample *t* test. The left column displays GM spatial maps, while the right column shows corresponding fMRI spatial maps. Spatial maps were *z*-scored and thresholded at |*Z*| > 2.5 and overlaid with anatomical images. The color bar represents the *z*-scored intensity value—this reflects relative structural or functional loadings. Warm colors (red/yellow) indicate regions with increased spatial expression in GM and fMRI in the AD group compared with those in the HC group, whereas blue indicate reduction in the AD group relative to that in the HC group. Each GM–fMRI pair captures a distinct network-specific structure–function coupling, consistent with the results summarized in Table 1.

Furthermore, we evaluated the spatial maps of these significant components to reveal their structure–function coupling. Here, coupling refers to co-occurrences of different regions within the same component, meaning that changes in one region (e.g., region A in GM) are systematically associated with changes in another region (e.g., region B in fMRI). For instance, region A in the GM map may structurally support or enable neural activation in region B in the fMRI map—or in some cases, the same region A may exhibit both structural and functional modulation. Unlike unimodal analysis, our pmg-jICA captures these two or multiple regions within a single component that tells that these regions are correlated, associated, or coupled. In component 2 of combination 1, shown in Figure 4, the GM spatial map shows spatial expression in the middle temporal gyrus of the auditory (AU) regions, which is increased in the AD group compared with that of the HC group. The corresponding fMRI spatial map highlights spatial expression in caudate (subcortical [SC] domain) and cognitive control (CC) domain. Among these, SC domain shows reduced spatial expression in the AD group compared with that of the HC group, whereas the CC domain exhibits both increased and decreased spatial expression, reflecting a complex pattern of functional alteration. In component 4 of combination 2, the GM spatial map shows decreased spatial expression in the insula and inferior frontal gyrus (CC) and superior temporal gyrus (AU) in the AD group compared with those of the HC group. The corresponding fMRI spatial map shows reduced spatial expression in the thalamus (SC) and lingual gyrus (VS) in the AD group, but some regions of CC nearly at hippocampal are functionally increased in the AD group compared with those of the HC group. The structure–function coupling of all the significant components are illustrated in Table 1. Coupling reveals the covarying regions in GM and fMRI, and group differences in spatial expression are assessed by comparing subject loadings in AD versus HC groups. One of the strengths of our approach is it can separate overlapping regions into different components, where one is subtractive and another one is additive, functionally and/or structurally.

**Table 1.** This table summarizes active regions of the statistically significant GM–fMRI component pairs identified through pmg-jICA

	**Modality**	**Brain networks**	**Inference**
Com-1: C-2	GM	AU: Middle temporal gyrus	Increased in AD
	fMRI	Areas of CC	Increased in AD
		SC: Caudate; areas of CC	Decreased in AD
Com-2: C-4	GM	AU: Middle temporal gyrus	Increased in AD
		CC: Insula, inferior frontal gyrus	Decreased in AD
	fMRI	CC: Nearly at hippocampal regions	Increased in AD
		SC: Thalamus; VS: Lingual gyrus	Decreased in AD
Com-3: C-6	GM	AU: Superior temporal gyrus	Decreased in AD
		SC: Thalamus, caudate	
		CC: Insula, inferior frontal gyrus	
	fMRI	AU: Superior temporal gyrus, middle temporal gyrus	Increased in AD
		SC: Caudate, putamen	Decreased in AD
		CC: Parahippocampal	
Com-4: C-6	GM	Areas of CC	Decreased in AD
	fMRI	Caudate, putamen	Decreased in AD
Com-20: C-8	GM	SC: Caudate, thalamus;	Increased in AD
		Areas of CB	
	fMRI	Some areas of VS	Increased in AD
		Some areas of VS	Decreased in AD
Com-23: C-8	GM	Areas of CB	Increased in AD
	fMRI	VS: Middle occipital gyrus	Increased in AD
		AU: Middle temporal gyrus	
Com-25: C-6	GM	AU: Superior temporal gyrus	Decreased in AD
		CC: Insula, Inferior frontal gyrus, parahippocampal gyrus	
	fMRI	AU: Middle temporal gyrus, inferior temporal gyrus	Increased in AD
		Areas of VS	Decreased in AD
Com-28: C-3	GM	AU: Superior temporal gyrus;	Decreased in AD
		SC: Thalamus, caudate	
		CB: Declive, uvula, tuber	
	fMRI	CC: Superior frontal gyrus, medial frontal gyrus	Increased in AD
		CC: Middle frontal gyrus	Decreased in AD
Com-42: C-3	GM	Areas of CB	Increased in AD
	fMRI	AU: Middle temporal gyrus	Increased in AD
		DMN: Posterior cingulate	
		CC: Middle frontal gyrus, medial frontal gyrus, supramarginal gyrus	Decreased in AD
Com-51: C-4	GM	Areas of CB	Increased in AD
	fMRI	Areas of VS	Decreased in AD
		Areas of CB	Increased in AD
Com-53: C-2	GM	AU: Inferior temporal gyrus; areas of CB	Increased in AD
	fMRI	Areas of VS; areas of CB	Increased in AD
		Areas of CC	Decreased in AD

Each combination highlights distinct network-specific alterations, indicating regions that show expression either increases or decreases in the AD group compared with that in the HC group. The activation of these multiple spatially related regions co-occur from both modalities within the same ICN, making coupling between GM and fMRI. An increase or decrease in GM and fMRI spatial expression refers to the AD group relatively to that of the HC. Abbreviations: Com = combinations; C = component; DMN = default mode network.

### FNC

**Figure 5.**
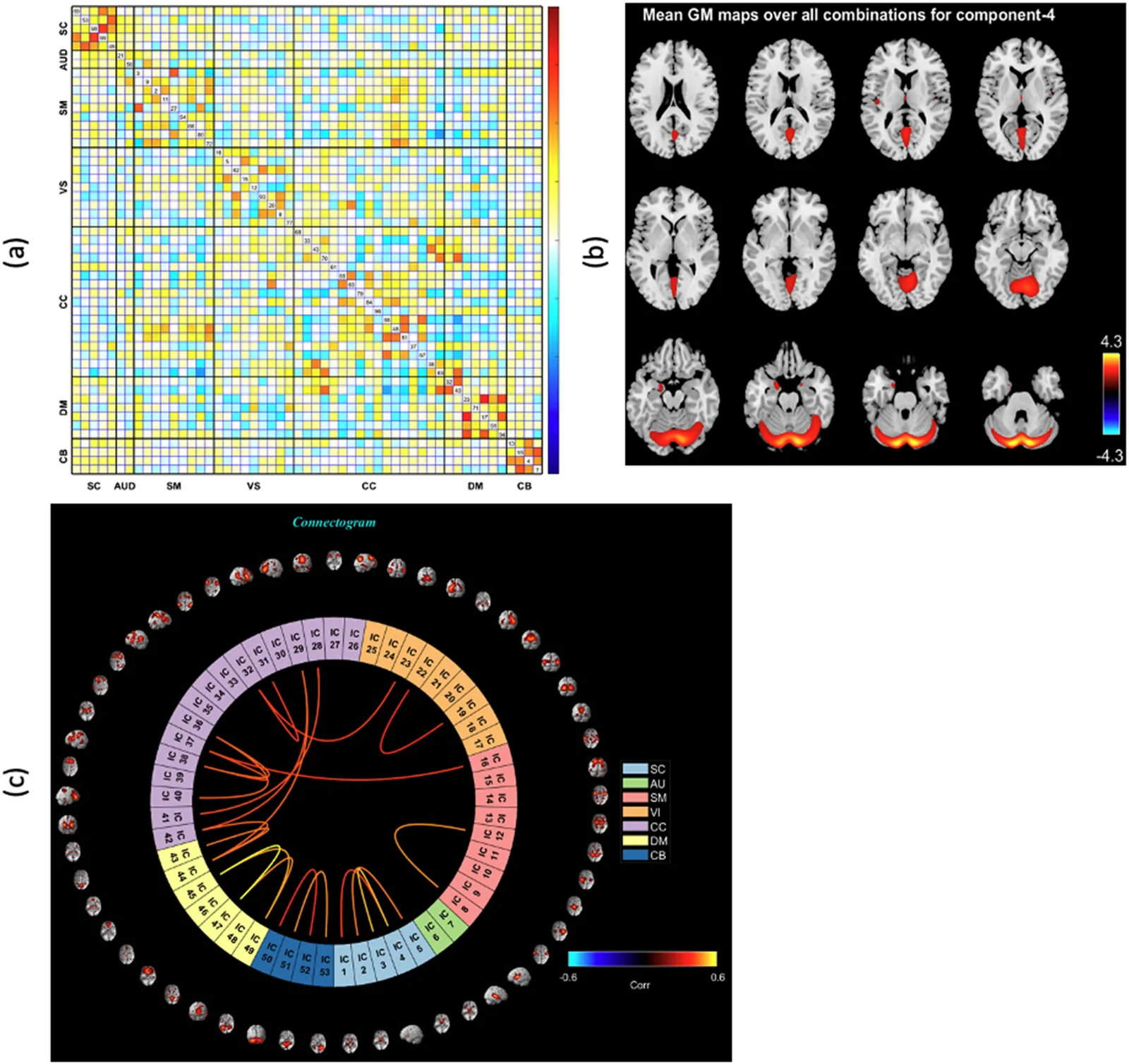
Joint component 4: (A) 53 × 53 FNC matrix derived from subject loadings, showing pairwise correlations among ICNs. Warm colors represent positive correlations, while cool colors indicate negative correlations. The FNC matrix represents a modular or blocked structure—clusters (modules) of networks that are more strongly connected to each other than other networks. For example, there is a strong within-network association in DM, CB, SC, and CC, along with fewer components of SM and VS networks. (B) Mean GM spatial component maps associated with component 4 across all network combinations, highlighting dominant contributions from VS and SC regions. These patterns reflect structural covariance within the component. (C) Connectogram summarizing significant FNC patterns at the network level, illustrating coordinated interactions between DM, CC, SC, and VS networks. This example was randomly selected to demonstrate the interpretability of the rich pmg-jICA outputs; all other components are presented in the Supporting Information Figures.

We computed 53 × 53 FNC matrices for 10 joint components and paired them with corresponding aggregated GM. The FNC matrix of joint component 4 (C-4) is depicted in Figure 5A. Positive functional internetwork connectivity within the default mode (DM), cerebellar (CB), SC, and CC, along with fewer components of sensorimotor (SM) and VS networks, has been observed in the FNC matrix. It reveals that these networks are positively correlated. Further connectogram in Figure 5C reveals positive connectivity between the hippocampus of the CC and the postcentral gyrus within the SM network. Furthermore, we identified positive connectivity between the precuneus of the DM network and the inferior frontal gyrus of the CC. Additionally, our findings revealed positive connectivity between the inferior occipital gyrus within the VS network and the inferior parietal lobule of the CC. The corresponding aggregated GM map shown in Figure 5B exhibits positive voxel weights in CB and VS regions, indicating areas that contribute positively to the GM component and covary with stronger functional connectivity within the same networks during rest. Together, these findings highlight strong structure–function coupling across domains. The FNC, connectogram, and their corresponding aggregated GMs for the rest of the joint components are presented in the Supporting Information section.

### Classification

**Figure 6.**
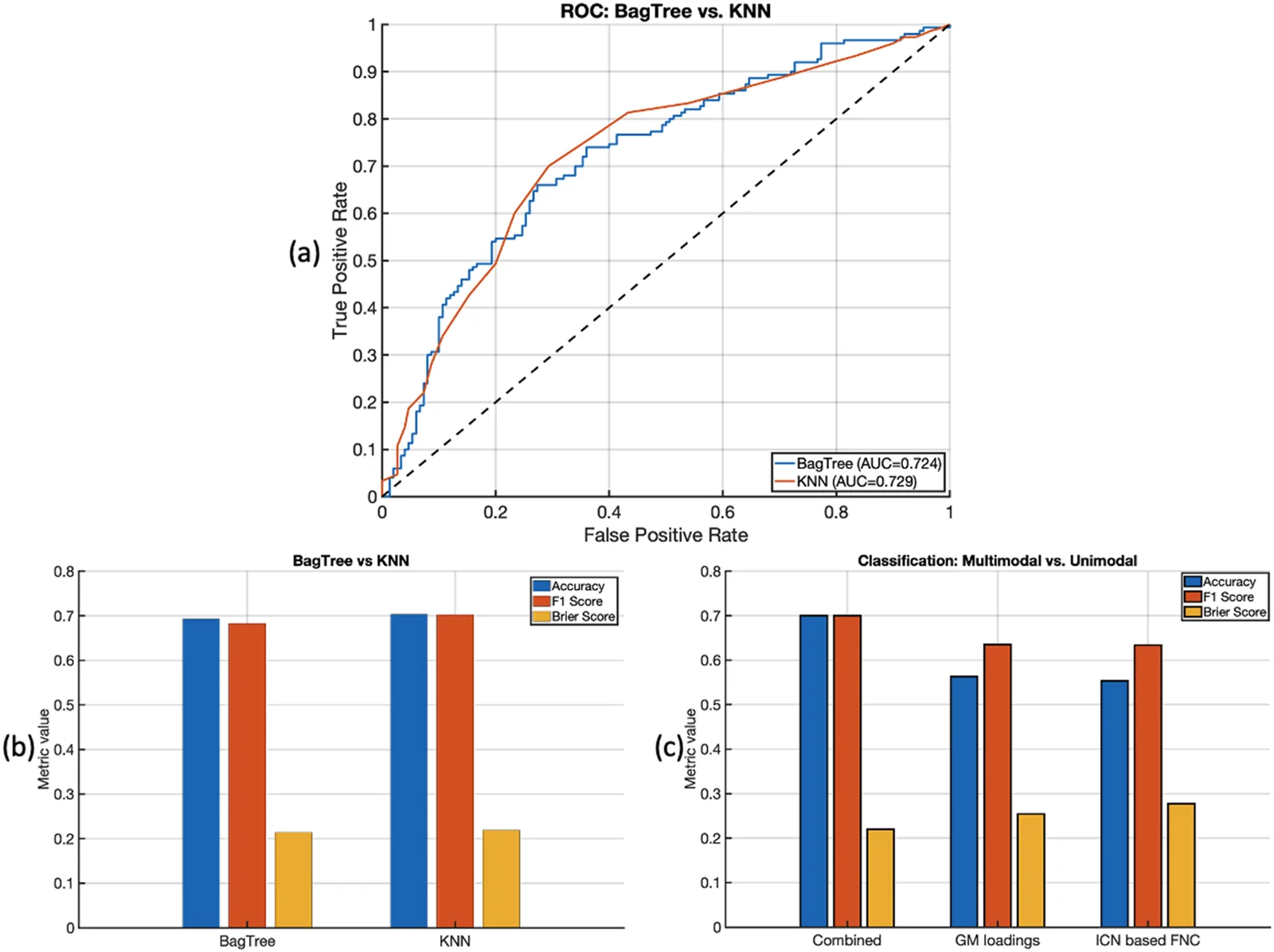
This figure displays the classification performance using unimodal and multimodal features derived from pmg-jICA. (A) ROC curves for BagTree and KNN classifiers using the combined multimodal features (GM loadings + ICN-based FNC). Both the classifiers show AUC around 0.72. (B) Comparison of classification metrics for BagTree and KNN, including accuracy, F1 score, and Brier score, demonstrating consistent performance across classifiers. (C) Comparison of classification performance across feature sets: combined multimodal features, GM loadings only, and ICN-based FNC only. The multimodal feature set consistently outperforms unimodal feature sets across metrics. All results are obtained using 10-fold cross-validation with pooled out-of-fold predictions.

Figure 6 summarizes the classification performance obtained using unimodal and multimodal features derived from the proposed pmg-jICA framework. Figure 6A shows ROC curves for BagTree and KNN classifiers using the combined multimodal feature set. The AUC is 0.72 for both the classifiers, indicating stable and consistent generalization across classifiers. The similarity of the ROC curves suggest that the observed performance is driven primarily by the underlying multimodal features rather than by a specific classifier choice. Figure 6B presents the classification accuracy, F1 score, and Brier score. Both classifiers achieve similar classification accuracy (approximately 70%–71%) and F1 score, with low Brier scores, indicating good probabilistic calibration. Figure 6C compares classification performance across multimodal and unimodal feature sets. The combined multimodal features (GM loadings + ICN-based FNC) consistently outperform both unimodal alternatives.

## DISCUSSION

In this study, we focused on integrating information from multiple brain networks and brain structure. Our earlier work (Khalilullah et al., 2023) was limited to fewer networks including posterior DM, CB, and SC. Here, we present a significant extension by expanding our focus to include all 53 ICNs that allow us to evaluate network-specific functions as well as internetwork connectivity information. This work presents a technique to fuse GM and multiple rest fMRI networks using a novel approach that combines concepts in group ICA and joint ICA. The inclusion of group ICA also facilitates the use of back-reconstruction to estimate spatial maps for individual brain networks, which was not part of our previous algorithm. These maps were derived from a functional–structural fusion algorithm, allowing for individual testing to assess group differences or associations with variables of interest in the AD group. In addition, we can compute the relationship among brain networks via FNC from the results. Notably, our joint analysis separates overlapping regions of the brain networks, which may show opposite correlation from one another. This provides an extremely rich set of multimodal and unimodal output that has not been available in prior approaches.

To demonstrate the proposed framework, we identified various structural and functional alterations between HC and AD patients. Altered patterns were especially notable in AU, VS, SC, CC, and CB regions—all of which have been consistently implicated in psychiatric and neurodegenerative disorders, particularly AD (Agosta et al., 2023; Azeem et al., 2023; Cai et al., 2015; Cheng et al., 2023; Elvira-Hurtado et al., 2023; Kawabata et al., 2022; Khalilullah et al., 2023; Kim et al., 2023; Lesh et al., 2011; Lin et al., 2017; Liu et al., 2018; Mavroudis, 2019; McEvoy et al., 2023; Sendi et al., 2023; Tang et al., 2021; Tarawneh et al., 2022; Tentolouris-Piperas et al., 2017; Xiao et al., 2023).

Our results reveal that SC regions (including thalamus, caudate, and putamen) show reduced spatial expression in both fMRI and GM components in the AD group compared with those of the HC group, indicating concurrent decreases in functional and structural expression. SC structures serve as critical sensory gates, relaying information to cortical areas, and their disruption may underlie widespread cortical dysfunction (Lei et al., 2018; Sherman, 2005). Prior studies have similarly reported SC abnormalities in AD and schizophrenia (Cai et al., 2015; Khalilullah et al., 2023; Kim et al., 2023; Lesh et al., 2011; Tentolouris-Piperas et al., 2017; Xiao et al., 2023). FNC analysis paired with mean GM (Figure 5) further suggests a positive association of the caudate nucleus and thalamus with multiple domains. It appears that the basal ganglia (BG) region may be more directly linked to each of these regions. Interestingly, this coordination of the SC region has not been reported before, exhibiting a new perspective on how BG may influence brain function in AD.

Additionally, we identified reduced spatial expression in both GM and fMRI in the AD group compared with that of the HC group involving CC regions. Specifically, we observed reduced GM spatial expression within the insula and inferior frontal gyrus in the AD group compared with those in the HC group, while the parahippocampal region, known for its role in memory, showed reduced functional spatial expression alongside reduced structural spatial expression in the AD group relative to that of the HC group. These findings are consistent with prior reports identifying CC regions, particularly the insula and inferior frontal gyrus, acting as vulnerable hubs in AD (Agosta et al., 2023; Lin et al., 2017; Liu et al., 2018; Schwab et al., 2020; Sendi et al., 2023).

Structural and functional changes were also evident in AU regions. The superior temporal gyrus showed reduced GM spatial expression in the AD group relative to that of the HC group, while the middle and inferior temporal gyrus shows increased functionally spatial expression in the AD group relative to that of the HC group. Such alterations may contribute to AU processing deficits commonly reported in AD patients (Azeem et al., 2023; Khalilullah et al., 2023; McEvoy et al., 2023; Tarawneh et al., 2022).

Within the VS network, the lingual gyrus and middle occipital gyrus showed reduced functional spatial expression in the AD group relative to that of the HC group, consisting with the prior studies (Elvira-Hurtado et al., 2023; Kawabata et al., 2022; Liu et al., 2017). We also observed structural–functional alternation in the lingual gyrus and the middle occipital gyrus of the VS network. The observed alteration in structure and function of the VS network in AD patients may contribute to cognitive deficits and give rise to various clinical symptoms associated with AD.

In CB regions, we observed both higher and lower structural and functional spatial expression in the AD group relative to that of the HC group, depending on the specific joint component. The cerebellum is interconnected with various brain regions through extensive neural networks. Alterations in the cerebellum may impact these functional networks and contribute to the overall cognitive decline seen in AD. Prior studies suggest that the cerebellum, traditionally associated with motor function, may undergo structural and functional changes in AD (Cheng et al., 2023; Mavroudis, 2019; Tang et al., 2021). Recent research show that the cerebellum contributes to attention and executive control, working memory, cognitive flexibility, visuospatial reasoning and planning, language, as well as emotional regulation and social cognition (Buckner, 2013; LeBel & D’Mello, 2023; Neudorf et al., 2019; Price, 2012; Zhang et al., 2023). Understanding the extent and implications of CB involvement is crucial for gaining a comprehensive understanding of the brain disorder and may open new avenues for therapeutic interventions.

Further classification results show that multimodal components estimated through pmg-jICA provide additional discriminative information beyond either modality. These results show that while pmg-jICA is not designed as an end-to-end predictive model, the subject loadings encode generalizable and discriminative information. The classification analysis therefore serves as a supporting validation of the proposed fusion framework, confirming that the identified structure–function couplings are not only neurobiologically interpretable but also informative at the individual-subject level. Finally, we note that achieving higher classification accuracy would likely require a different modeling strategy, including higher-dimensional feature representations and end-to-end optimization for prediction. Incorporating such predictive objectives into the fusion model itself represents an important direction for future work.

In contrast, the pmg-jICA solution revealed more complete and spatially coherent multimodal components, including structure–function couplings that did not appear in either unimodal solution. For instance, in component 4 of combination 2, the GM map involved CC and AU networks, whereas the corresponding fMRI map expressed CC and SC patterns. This coordinated multimodal expression was unique to the joint model, highlighting mechanistic links across modalities that are invisible in unimodal analyses. The subject loadings analysis also showed that multimodal integration enhances sensitivity to AD-related alterations. Pmg-jICA identified 11 components with significant AD–HC differences across CC, AU, SC, VS, DM, and CB networks. By contrast, unimodal fMRI ICA yielded significant group differences only in SC and VS components. The broader and more robust discriminative profile of pmg-jICA underscores the importance of integrating structure and function when characterizing distributed neurodegenerative processes.

Although, the findings are promising, there are several limitations of the proposed pmg-jICA framework. First, the method assumes spatial correspondence after registration to MNI space, which may lead to misalignment between GM and fMRI networks, could influence joint component estimation. Second, the current study used the NeuroMark 1.0 template to generate 53 ICNs; however, the method has not yet been evaluated with the updated NeuroMark 2.0 templates. Future work may compare the joint components derived from both versions to assess reproducibility and potential improvements. Finally, pmg-jICA is currently designed to integrate sMRI and fMRI; extending it to other modalities such as PET, EEG, or diffusion MRI would be valuable but may require additional methodological adjustments.

Taken together, our findings provide detailed spatial maps and connectivity profiles that highlight network-level disruptions in AD. The proposed pmg-jICA framework extends the earlier pml-jICA model by introducing a group-level, multinetwork design. While pml-jICA links a single fMRI network with GM through a global joint component, pmg-jICA fuses multiple ICNs separately and then integrate them across subjects. This design allows multiple networks to interact and reveals how structural and functional features jointly vary across the brain. Importantly pmg-jICA incorporate pml-jICA within its joint ICA decomposition stage—using it as core algorithm for estimating joint components. However, pmg-jICA extends beyond a single global mapping by repeating this decomposition across multiple ICNs and integrating the results at the group level. This hierarchical fusion design allows pmg-jICA to capture network-specific, subject-specific, and internetwork structure–function relationship that pml-jICA cannot capture. This multimodal fusion framework not only extends prior work but also offers a rich platform for exploring localized and network-level abnormalities in neurodegenerative disease.

## CONCLUSIONS

In conclusion, our study presents a robust framework, pmg-jICA, for the joint analysis of multimodal MRI data, enabling the fusion of structural and functional information. By overcoming challenges in integrating different imaging modalities, our approach enhances the depth of understanding of brain alterations in AD, by linking together and jointly decomposing data while still preserving the richness of the information available. The investigation into structural and functional alterations between healthy controls and Alzheimer’s patients highlights notable patterns in regions such as the AU, VS, SC, CC, and cerebellum. By conducting a joint analysis with group ICA, it facilitates the comprehensive utilization of the variability among structural connections to specific functional networks, and we can leverage the combined distribution of multimodal imaging data, ultimately enhancing our capability to discern between health and disease. The identified changes in connectivity and structure provide insights into the impact of AD on essential brain functions and behaviors. Moreover, the findings emphasize the importance of multimodal approaches in unraveling complex relationships that may remain elusive in unimodal studies. The study’s contributions extend beyond AD, offering a methodological advancement for the joint analysis of diverse neuroimaging data to enhance our understanding of brain disorders.

There are several possible future directions that could be considered for this work. Methodologically, the algorithm could be improved by integration of the temporal information captured by fMRI to capture a larger scope of brain dynamics, or by integrating additional modalities such as diffusion-weighted MRI to better study linkages of brain functional networks to white matter structure. Additionally, incorporating improved machine learning and deep learning approaches into the model architecture could help further optimize the integration of the multimodal information. Methodologically, future extensions of pmg-jICA will investigate the impact of alternative normalization strategies, including the use of unstandardized ICN maps, on component estimation. Importantly, the model can also be extended to subject-level prediction with high-dimensional feature representations and supervised learning algorithms. Future extensions will evaluate classification performance and generalizability. Future work could also focus on broadening the application of pmg-jICA across additional cohorts and in different patient populations, especially those with complex and heterogenous presentations with strong structural and functional elements including attention-deficit hyperactivity disorder (ADHD), autism, and schizophrenia. Moreover, application of pmg-jICA in a large, transdiagnostic dataset containing subjects with both neurodegenerative (i.e., AD) and neurodevelopmental (i.e., ADHD, autism) conditions could help to elucidate more of the structural and functional etiologies contributing to these two classes of conditions.

## ACKNOWLEDGMENTS

This study was supported by National Science Foundation 2316421 and 2112455 and National Institutes of Health RF1AG063153.

## SUPPORTING INFORMATION

Supporting information for this article is available at https://doi.org/10.1162/NETN.a.575.

## AUTHOR CONTRIBUTIONS

K. M. Ibrahim Khalilullah: Conceptualization; Data curation; Formal analysis; Investigation; Methodology; Validation; Visualization; Writing – original draft. Souvik Phadikar: Conceptualization; Data curation; Formal analysis; Investigation; Methodology; Validation; Visualization; Writing – original draft; Writing – review & editing. Oktay Agcaoglu: Methodology; Writing – review & editing. Jing Sui: Methodology; Writing – review & editing. Marlena Duda: Conceptualization; Methodology; Writing – review & editing. Tülay Adali: Methodology; Supervision. Vince D. Calhoun: Conceptualization; Funding acquisition; Investigation; Methodology; Project administration; Resources; Supervision; Validation; Writing – review & editing.

## FUNDING INFORMATION

Vince D. Calhoun, National Science Foundation (https://dx.doi.org/10.13039/100000001), Award ID: 2316421. Vince D. Calhoun, National Science Foundation (https://dx.doi.org/10.13039/100000001), Award ID: 2112455. Vince D. Calhoun, National Institute of Health, Award ID: RF1AG063153.

## DATA AND CODE AVAILABILITY

The data are used from the OASIS-3 and can be access via the following website: https://www.oasis-brains.org. The pmg-jICA method and example run code can be found in the FIT toolbox at https://github.com/trendscenter/fit.

## Supplementary Material



## TECHNICAL TERMS

Structure–function coupling: The relationship between brain anatomy and functional activity across regions or networks.
Gray matter (GM): Brain tissue containing neuronal cell bodies, responsible for information processing.
Parallel multilink group joint ICA (pmg-jICA): A multimodal fusion framework linking structural MRI with multiple functional networks in a unified group model.
Independent component analysis (ICA): A statistical technique that separates mixed signals into independent underlying patterns.
Joint ICA (jICA): A multimodal method that decomposes different imaging modalities simultaneously to identify linked components.
Group ICA: An ICA framework that identifies common brain components shared across multiple subjects.
Functional network connectivity (FNC): Statistical relationships between brain networks reflecting coordinated activity patterns.
Principal component analysis (PCA): A dimensionality reduction technique that summarizes large datasets into fewer dominant patterns.
Intrinsic connectivity network (ICN): A group of brain regions that show synchronized activity during rest.
Dual regression: A method used to estimate subject-specific brain maps from group-level components.
