# Supplementary material for: Parallel multilink group joint ICA (pmg-jICA): Fusion of 3D structural and 4D functional data across multiple resting fMRI networks
Source: Netw Neurosci. 2026 Aug 25;10(3):787–808. doi: 10.1162/NETN.a.575 (PMC13569335; doi:10.1162/NETN.a.575)
Supplement: Supplementary file 1 [file netn-10-3-787-s001.pdf]

The demographic and clinical details of the refined experimental data is presented in Table I.

Table I: Demographics and clinical information of the dataset.

**Subset**	**Metric**	**Count/Range**
**Full Data**	Total N (HC/AD)	260 (130/130)
**HCs**	Gender (M/F)	54/76
	Age at entry (years)	42.45-94.93
	CDR Score (range)	0-0
	Sum of Boxes (SOB)	0.0-0.5
**ADs**	Gender (M/F)	68/62
	Age at entry (years)	50.29-95.57
	CDR Score (range)	0.5-2.0
	SOB	0.5-12.0

Regression based reconstruction

Given the ICA decomposition in equation Eq. 5, regression analysis can be expressed as:

$$\widehat{\boldsymbol{X}}_k^T = \boldsymbol{S}^T \boldsymbol{L}_k^T + \varepsilon_k^T \tag{13}$$

Using ordinary least squares, $\boldsymbol{L}_k^\top$ can be expressed as:

$$\boldsymbol{L}_k^\top = (\boldsymbol{S}\boldsymbol{S}^\top)^{-1}\boldsymbol{S}\widehat{\boldsymbol{X}}_k^\top \tag{14}$$

which can be equivalently written in terms of the pseudoinverse of $\boldsymbol{S}$:

$$\boldsymbol{L}_k^\top = \widehat{\boldsymbol{X}}_k \boldsymbol{S}^+, \; where\; \boldsymbol{S}^+ = \boldsymbol{S}^\top(\boldsymbol{S}\boldsymbol{S}^\top)^{-1} \tag{15}$$

This first regression step can be interpreted as projecting the joint data matrix $\widehat{\boldsymbol{X}}_k$ onto the column space of $\boldsymbol{S}$: the product $\boldsymbol{L}_k\boldsymbol{S}$ is the orthogonal projection of $\widehat{\boldsymbol{X}}_k$ onto the subspace spanned by the aggregate spatial maps. In the second step, we estimated network-specific joint components $\boldsymbol{S}_k$ using the subject loadings. Given

$$\widehat{\boldsymbol{X}}_k = \boldsymbol{L}_k\boldsymbol{S}_k + \varepsilon_{2k}, \qquad E[\varepsilon_{2k}] = 0 \tag{16}$$

the least-squares estimate of $\boldsymbol{S}_k$ is

$$\boldsymbol{S}_k = (\boldsymbol{L}_k^\top\boldsymbol{L}_k)^{-1}\boldsymbol{L}_k^\top\widehat{\boldsymbol{X}}_k = \boldsymbol{L}_k^+\widehat{\boldsymbol{X}}_k \tag{17}$$

where $\boldsymbol{L}_k^+$ denotes the Moore–Penrose pseudoinverse of $\boldsymbol{L}_k$.It corresponds to the orthogonal projection of $\widehat{\boldsymbol{X}}_k$ onto the column space of $\boldsymbol{L}_k$. This two-step regression procedure constitutes the dual regression–type back-reconstruction, enabling ICN-specific joint spatial maps and subject loadings to be estimated from the aggregate joint ICA solution.

The component – 8, shown in Fig. 7, the SC, VS, and CB regions exhibit the most prominent within-network FNC, while the CC network displays a mixture of connections within intra and inter-networks. In inter-domain connectivity, we found increased FNC between component inferior parietal lobule and superior medial gyrus of CC and precuneus of DM. Furthermore, we identified positive connection between inferior parietal lobule from CC and middle temporal gyrus within AU. The subcortical, cerebellum regions of GM are also structurally connected. Although there isn't much cross-domain connectivity observed in these figures, the convergence of positive connectivity in both FNC and GM spatial maps for the SC, visual, and cerebellum suggests a strong relationship between the functional and structural aspects of these brain regions. Furthermore, the activation of SC, visual, and cerebellar regions in both GM and fMRI data implies a significant role of the basal ganglia (BG) in facilitating connections across multiple cognitive domains.

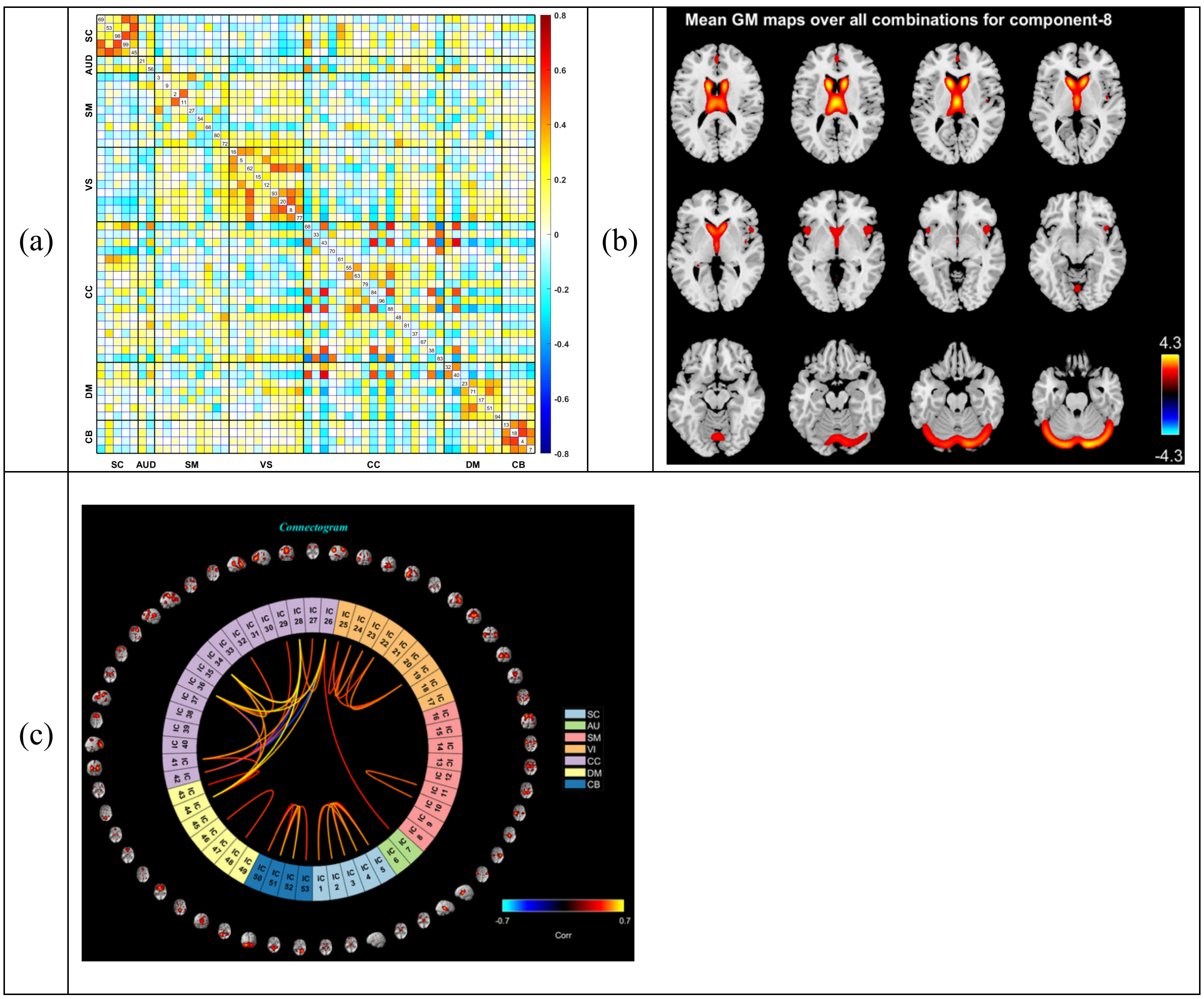

Fig. 7: Component – 8: (a) FNC matrix & (c) connectogram display the highly correlated regions are - precuneus, middle temporal gyrus, inferior parietal lobule, superior medial frontal gyrus, supplementary motor area, middle frontal gyrus, inferior frontal gyrus and SC domain. (b) represents the mean GM maps showing peak activity at SC, VS, and CB regions.

The component – 9, shown in Fig. 8, shows strong intra-network connectivity in CB, SC, and VS domain. There isn't extensive cross-connection, except for that observed between the precuneus of the DM and the inferior frontal gyrus originating from the CC. The GM spatial maps, shown in Fig. 7(b) also exhibits positive activity in CB, VS, and DM.

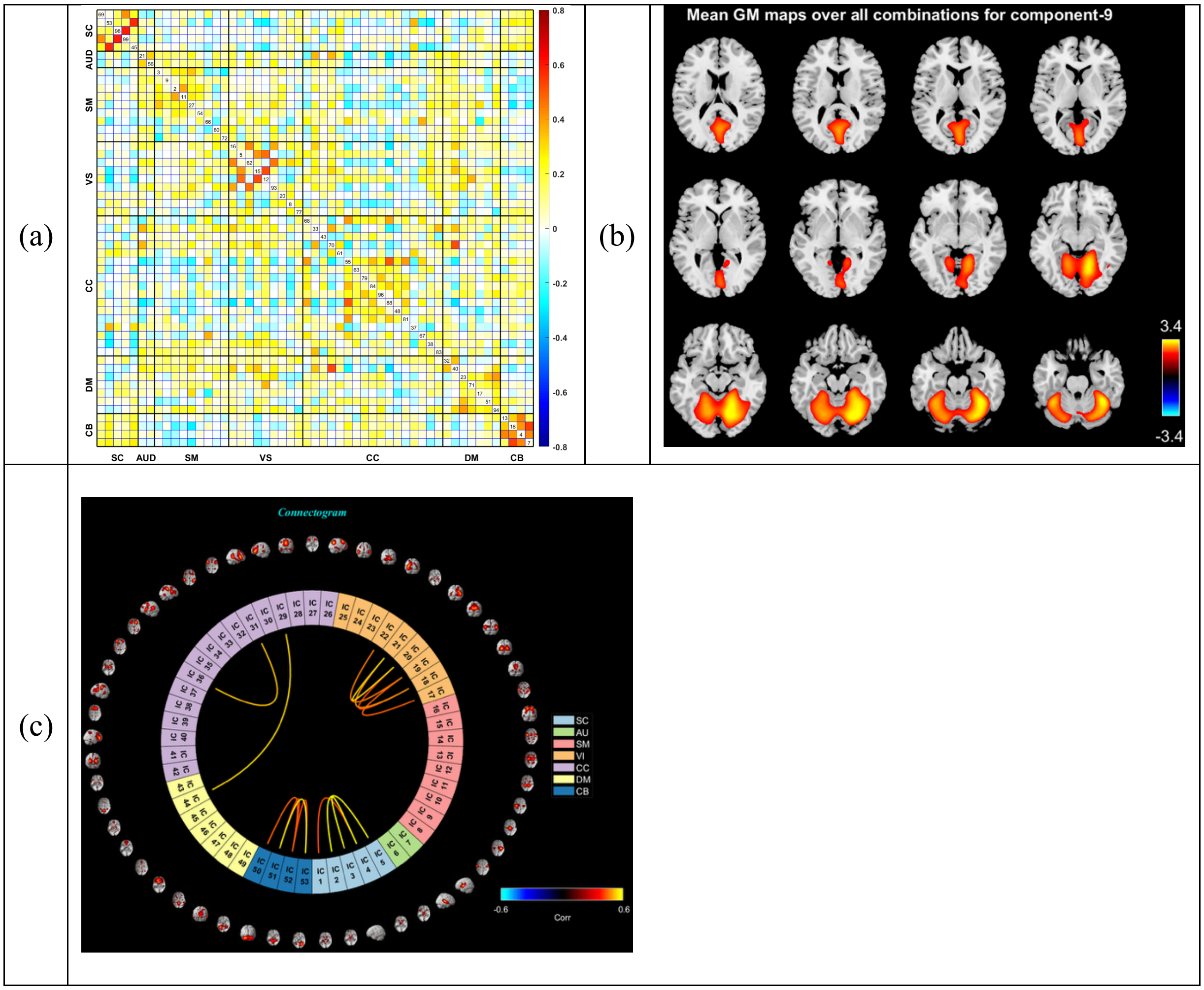

Figure 8: Component – 9: (a) FNC matrix and (c) connectogram reveals highly correlated regions are – hypothalamus, thalamus, superior temporal gyrus, middle occipital gyrus, middle cingulate cortex, insula, inferior frontal gyrus, middle frontal gyrus, posterior cingulate cortex, precuneus, and precentral gyrus. (b) represent mean GM maps for component 9 shows peak activation in DM, VS, and CB regions.

The component – 10, shown in Fig. 9, illustrates positive connectivity in SC, CB, SM, along with a combination of some highly correlated positive and negative connections in cognitive control. The GM maps also structurally increased in CC, SM, AU, and CB.

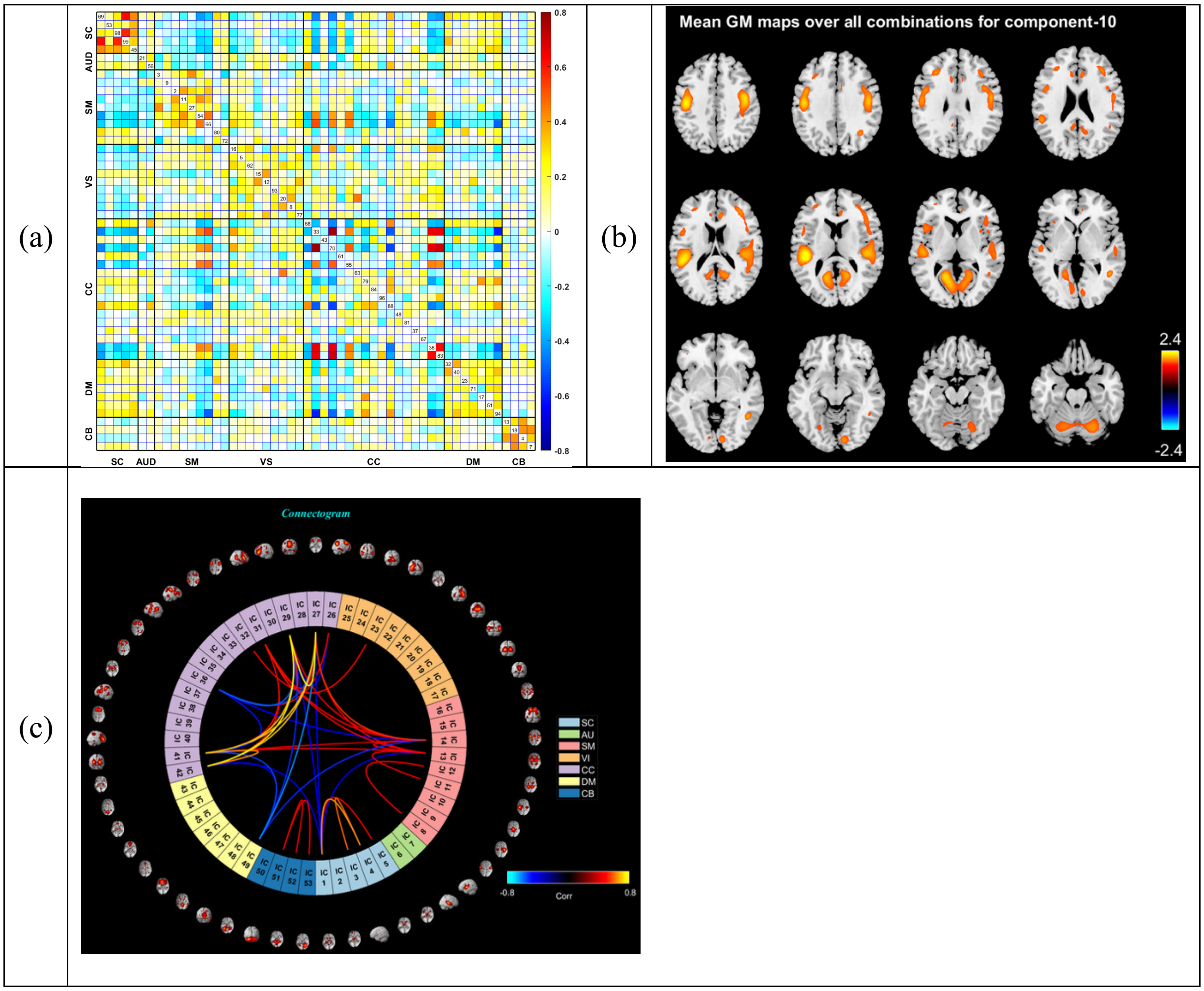

Figure 9: Component – 10: (a) FNC matrix and (c) connectogram reveals highly correlated regions are – anterior cingulate cortex, posterior cingulate cortex, insula, inferior frontal gyrus, middle frontal gyrus, hippocampus, precentral gyrus, and paracentral lobule. (b) represent mean GM maps for component 10 shows peak activation in CC, SM, and CB regions.

The component – 1, shown in Fig. 10, FNC shows increased connectivity within SC, VS, CC, and CB. The SC and CC components are more activated than VS and CB. In inter-network connectivity, we observed heightened connectivity between hippocampus from CC and postcentral gyrus within SM, between anterior DM and superior parietal lobule from SM. The GM map shows increased connectivity in AU, VS, and CB.

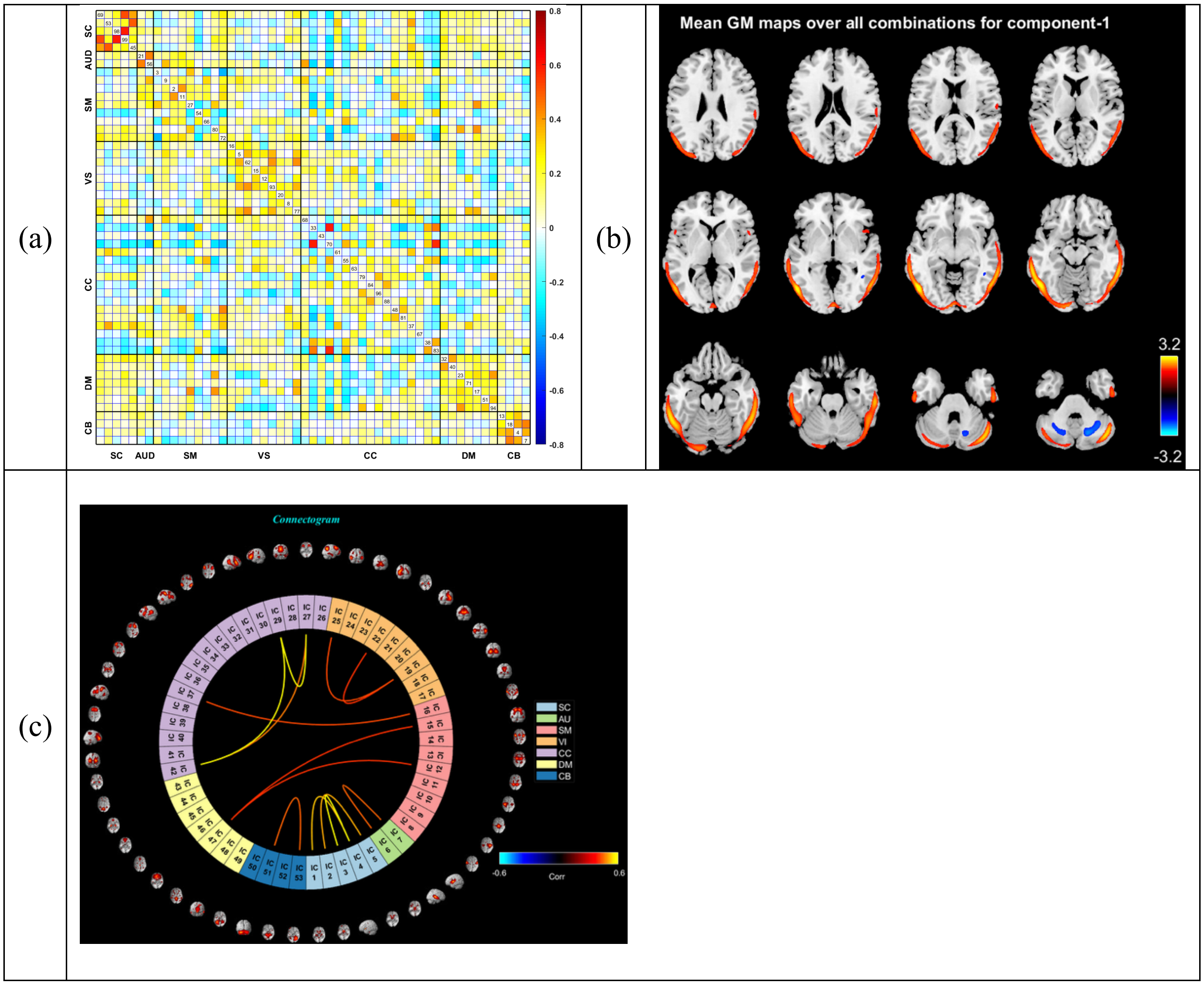

Figure 10: Component – 1: (a) FNC matrix and (c) connectogram reveals highly correlated regions are – hypothalamus, thalamus, caudate, middle cingulate cortex, middle temporal gyrus, inferior parietal lobule, insula, inferior frontal gyrus, anterior cingulate cortex, and superior parietal lobule. (b) represent mean GM maps for component 1 shows peak activation in AU, VS, and CB regions.

The component – 2, shown in Fig. 11, exhibits more activated regions in SC and VS within the networks. We also identify inter-network connectivity between hippocampus from CC and postcentral gyrus within SM; between inferior frontal gyrus from CC and putamen within SC; between middle occipital gyrus from VS and postcentral gyrus within SM. GM exhibits positive pattern in VS, AU, and CB.

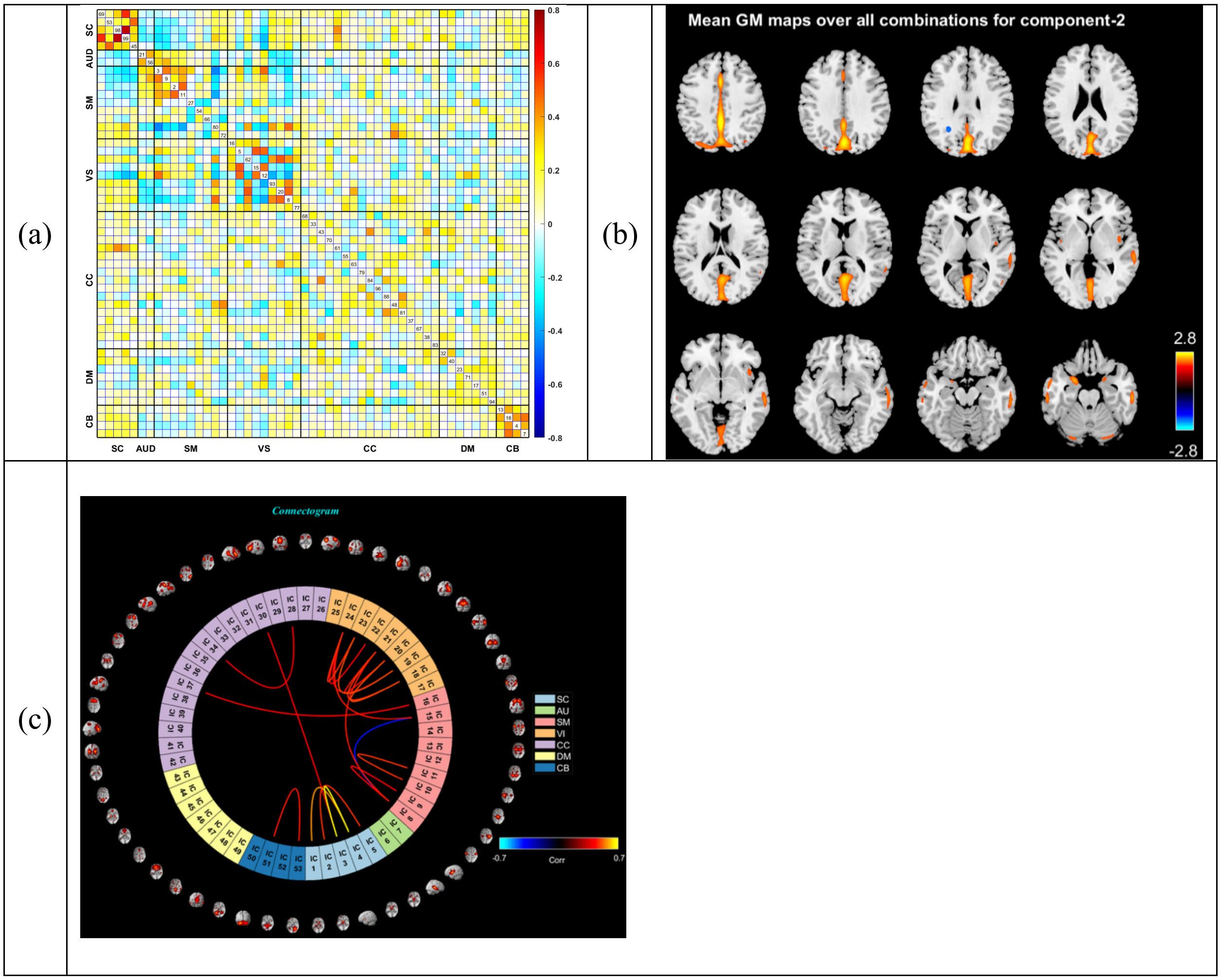

Figure 11: Component – 2: (a) FNC matrix and (c) connectogram reveals highly correlated regions are – hypothalamus, thalamus, caudate, middle cingulate cortex, hippocampus, left inferior parietal lobule, superior parietal lobule, postcentral gyrus, lingual gyrus, and middle temporal gyrus. (b) represent mean GM maps for component 2 shows peak activation in AU, VS, and CB regions.

The component – 3, shown in Fig. 12, exhibits highly positive connectivity regions in SC and SM, while SC shows a mixture of connections. In inter-network connectivity, there are increased connections between superior parietal lobule from AM, middle occipital gyrus within VS and anterior DM; between inferior parietal lobule, superior medial frontal gyrus from CC and middle temporal gyrus; between superior medial frontal gyrus, supplementary motor area from CC and anterior DM. The GM map exhibits positive activity in CB, SC and observed negative activation in BG regions.

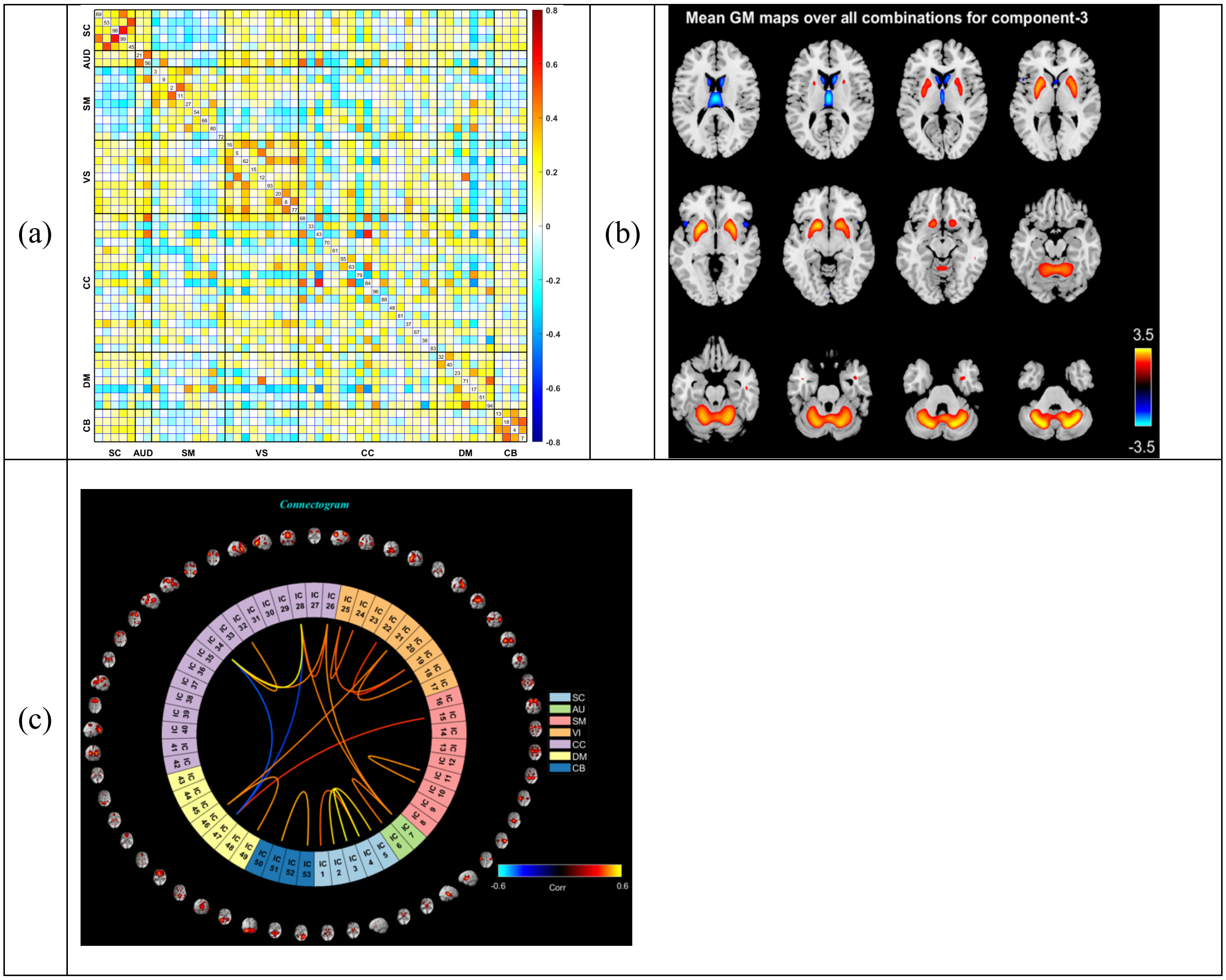

Figure 12: Component – 3: (a) FNC matrix and (c) connectogram reveals highly correlated regions are – inferior parietal lobule, insula, superior frontal gyrus, superior medial frontal gyrus, supplementary motor area, middle temporal gyrus, postcentral gyrus, superior parietal lobule, middle occipital gyrus fusiform gyrus, posterior cingulate cortex, anterior cingulate cortex, and SC domain. (b) represent mean GM maps for component 3 shows peak activation in CB, SC, and BG regions.

The component – 5, shown in Fig. 13, exhibits increased functional connectivity within SC and VS, they are more activated than other regions such as SM, CB, DM, and CC. In addition to intra-network connectivity, we identify increased connectivity between inferior frontal gyrus of CC and precuneus within DM; between hippocampus of CC and postcentral gyrus from SM; between precuneus of DM and hypothalamus of SC. The mean GM shows peak activation in VS and CB.

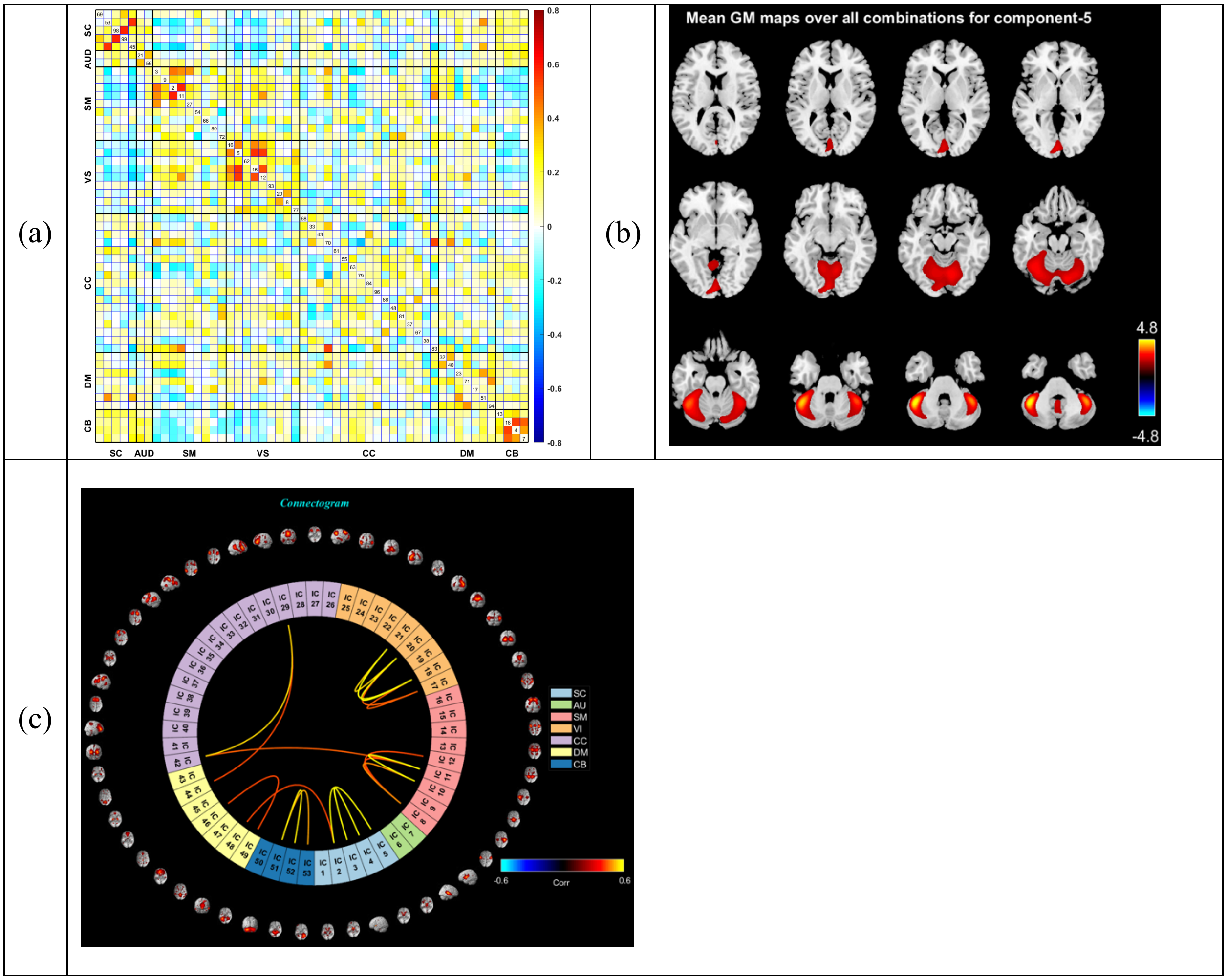

Figure 13: Component – 5: (a) FNC matrix and (c) connectogram reveals highly correlated regions are – precuneus, posterior cingulate cortex, inferior frontal gyrus, hippocampus, postcentral gyrus, paracentral lobule, middle occipital gyrus, middle temporal gyrus, cuneus, thalamus, hypothalamus, putamen. (b) represent mean GM maps for component 5 shows peak activation in CB and VS regions.

The component – 6, shown in Fig. 14, shows increased intra-network connectivity in SC, VS, SM, CB, CC, and DM. The SC and VS are highly activated than others, while there is a mixture connection within VS. In inter-network connectivity, we found increased connectivity between inferior frontal gyrus of CC and precuneus from DM; between inferior parietal lobule, inferior frontal gyrus from CC and inferior occipital gyrus within VS; between posterior DM and inferior occipital gyrus from VS. The mean GM shows peak activation in AU and SC, which is negative and implies that these regions are negatively correlated with observed functional connectivity.

(a)

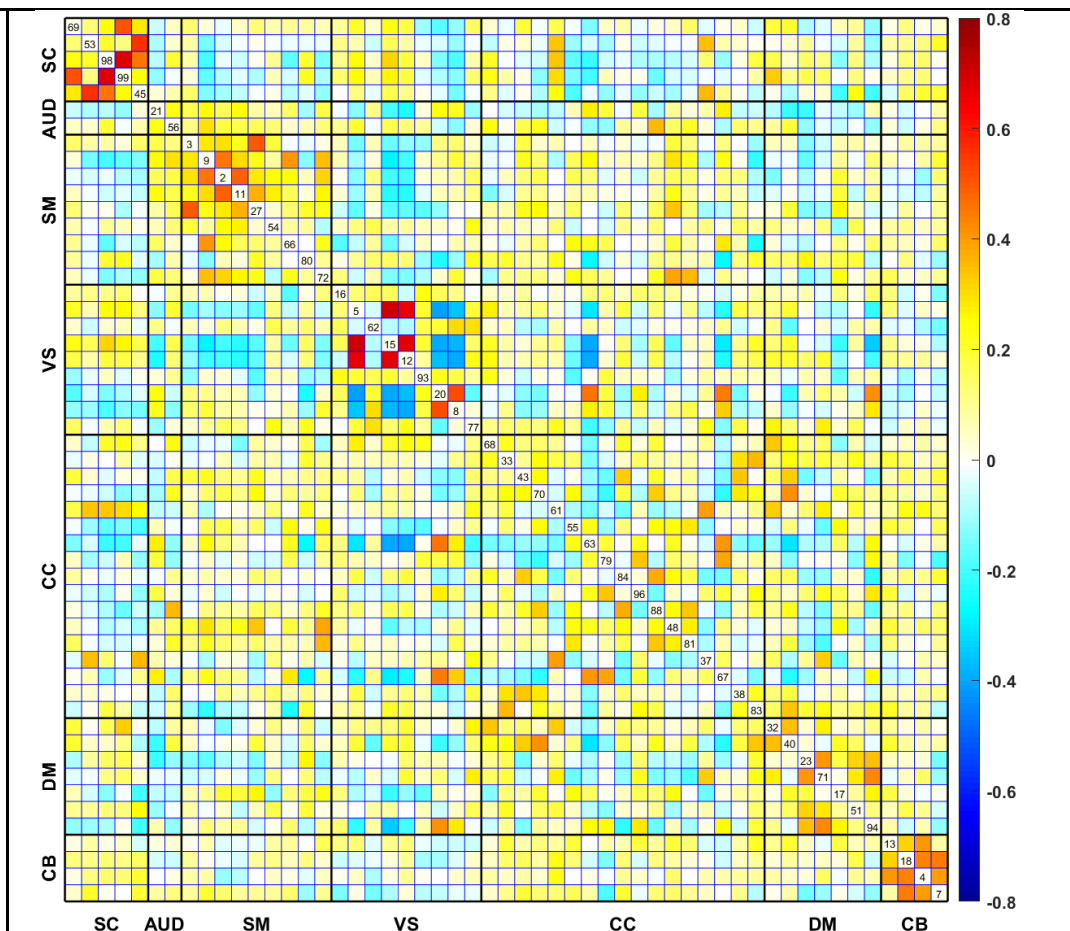

(b)

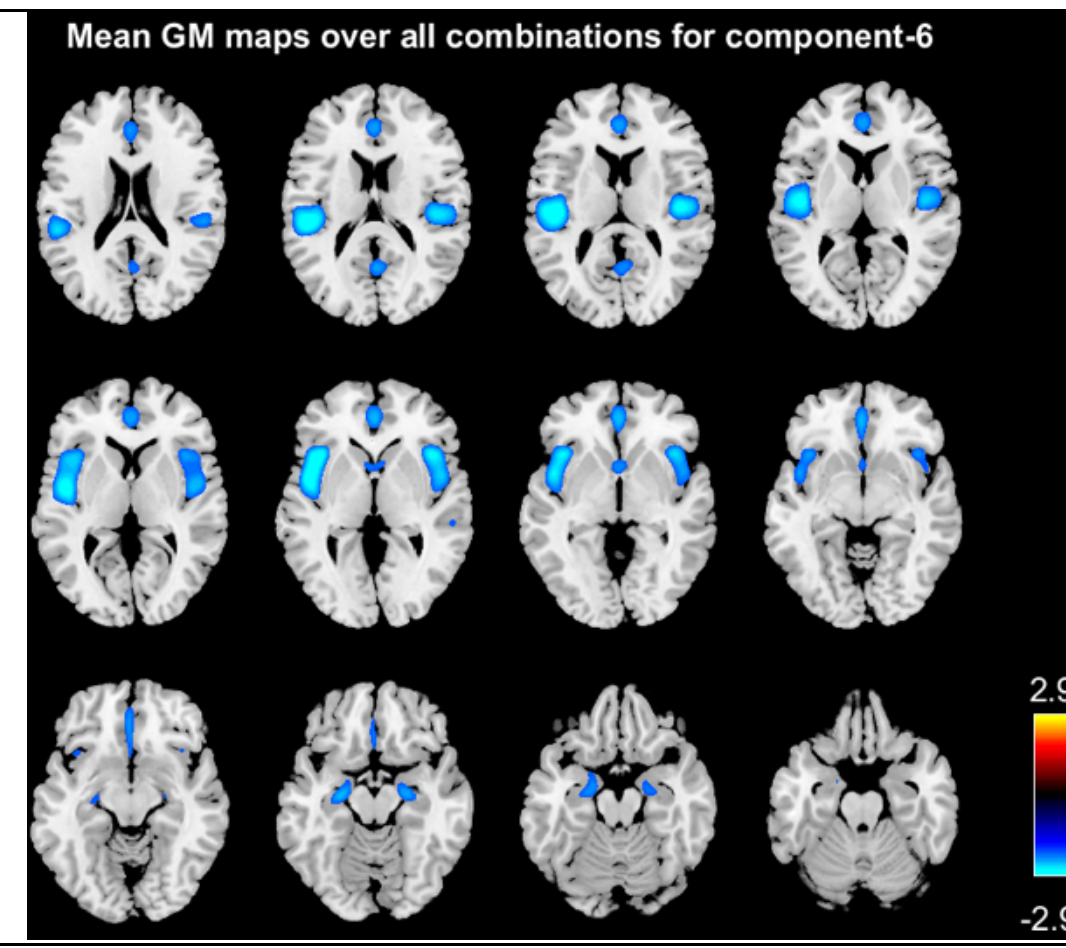

(c)

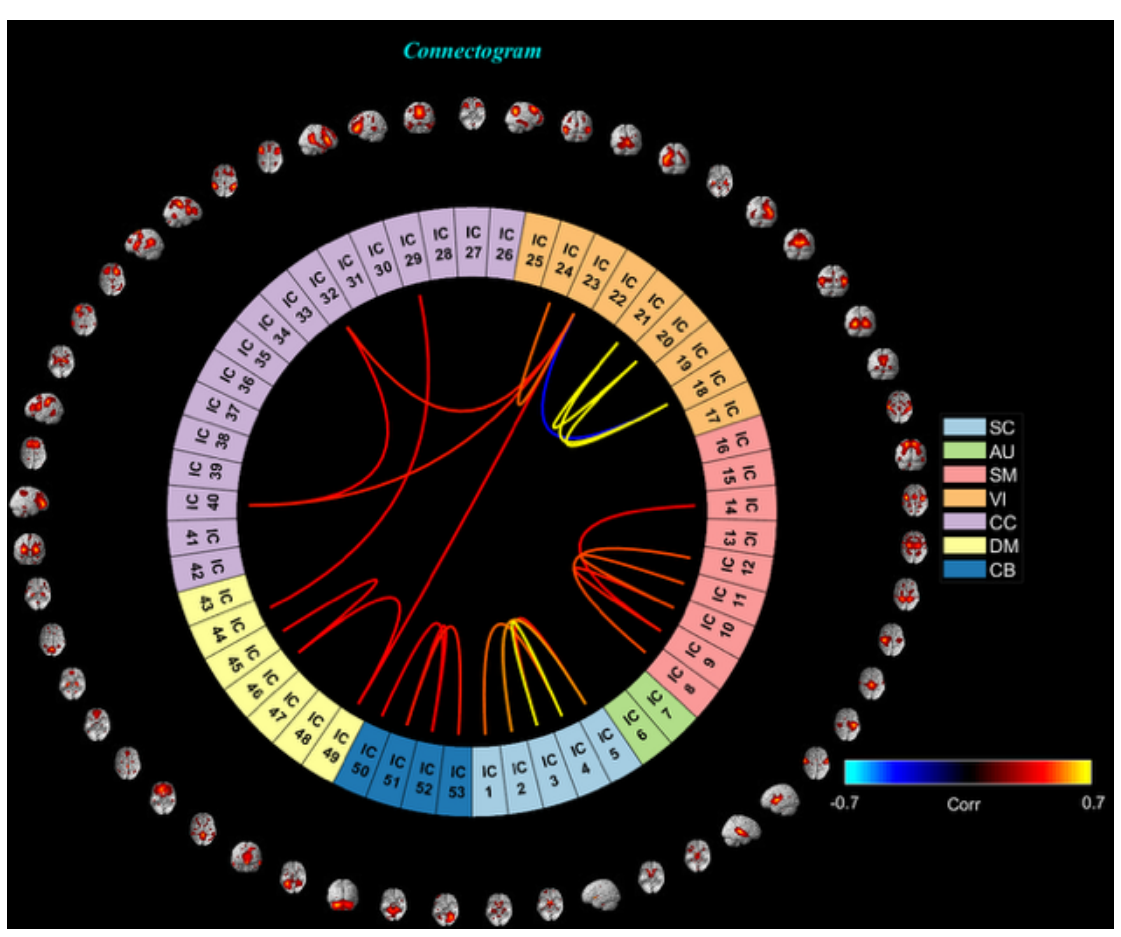

Figure 14: Component – 6: (a) FNC matrix and (c) connectogram reveals highly correlated regions are – hippocampus, left inferior parietal lobule, inferior frontal gyrus, posterior cingulate cortex, postcentral gyrus, inferior occipital gyrus, lingual gyrus. (b) represent mean GM maps for component 6 shows negative peak activation in AU and SC.

The component – 7, shown in Fig. 15, shows increased intra-network connectivity within SC, VS, also in SM and CC. We also observed inter-network connectivity between superior parietal lobule within SM, fusiform gyrus in VS and anterior DM; between Hippocampus from CC and right postcentral gyrus within SM; between left inferior parietal lobule from CC and postcentral gyrus within SM. The mean GM shows positive activation in CB and DM.

(a)

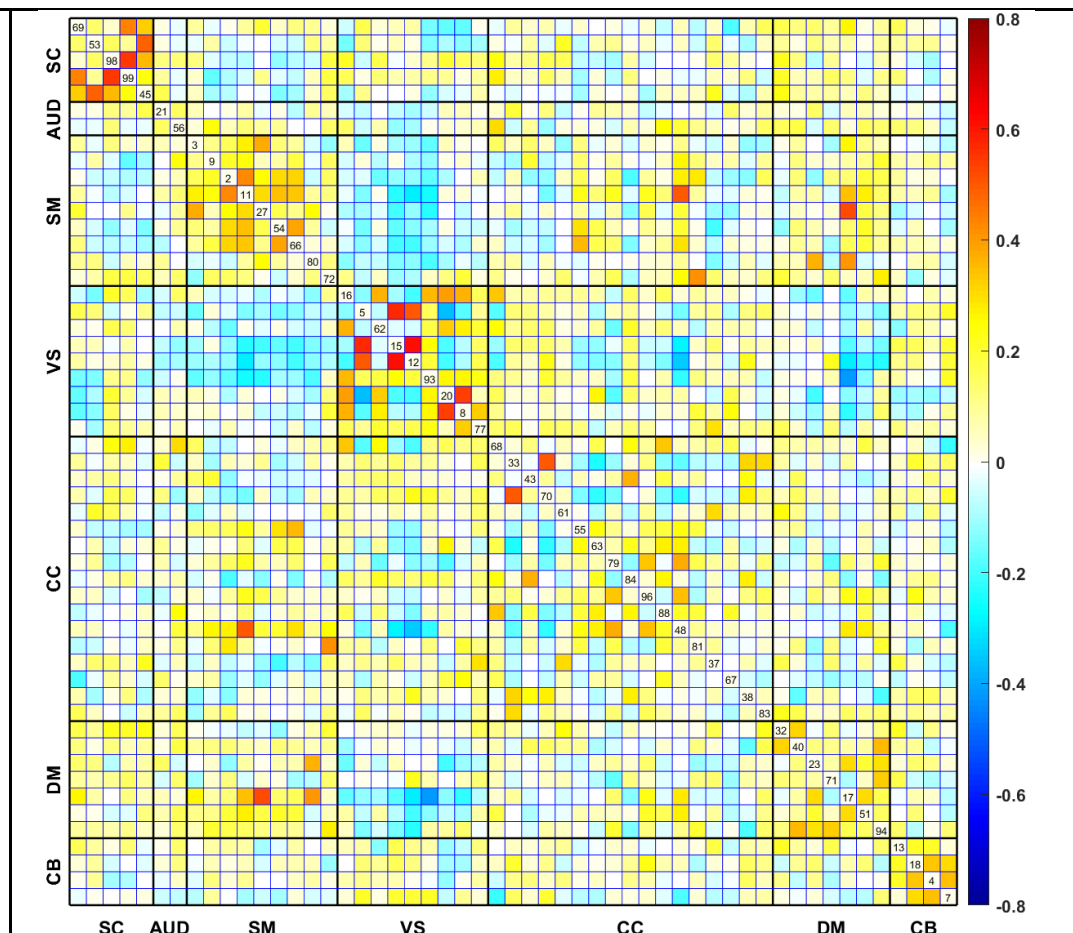

(b)

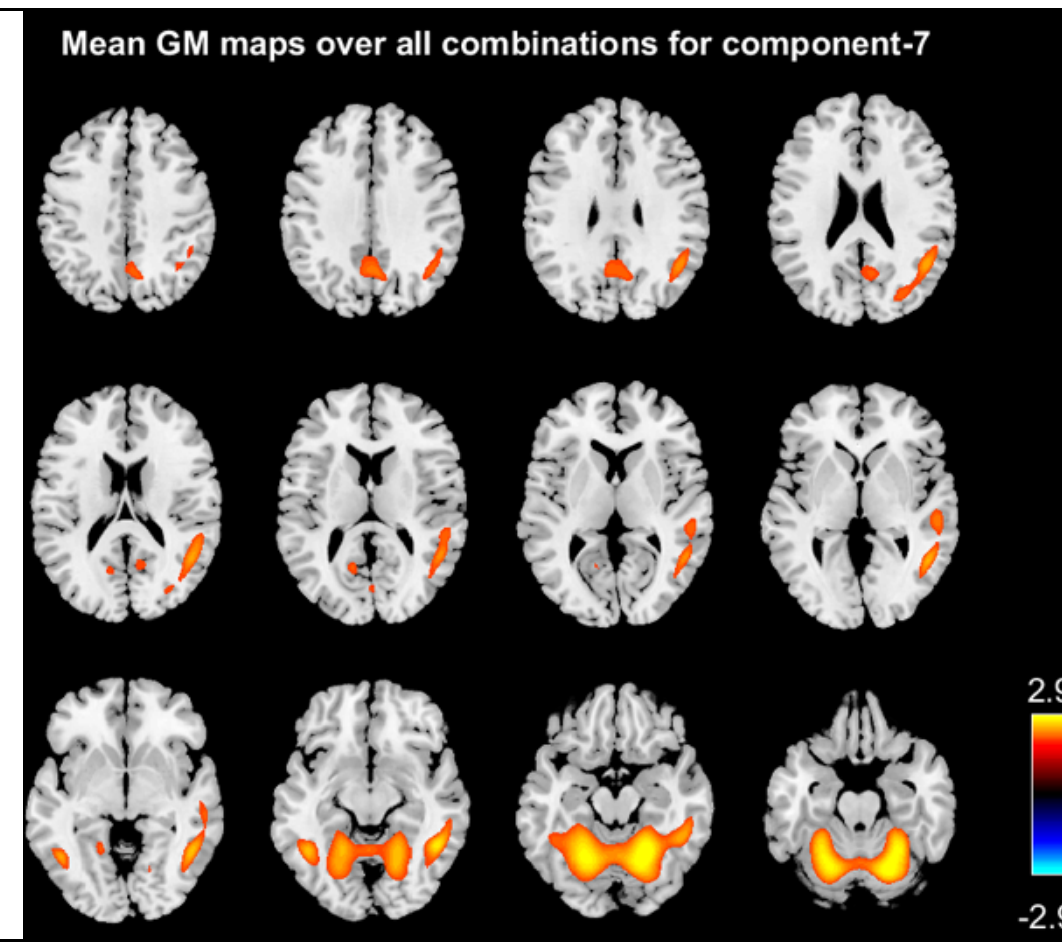

(c)

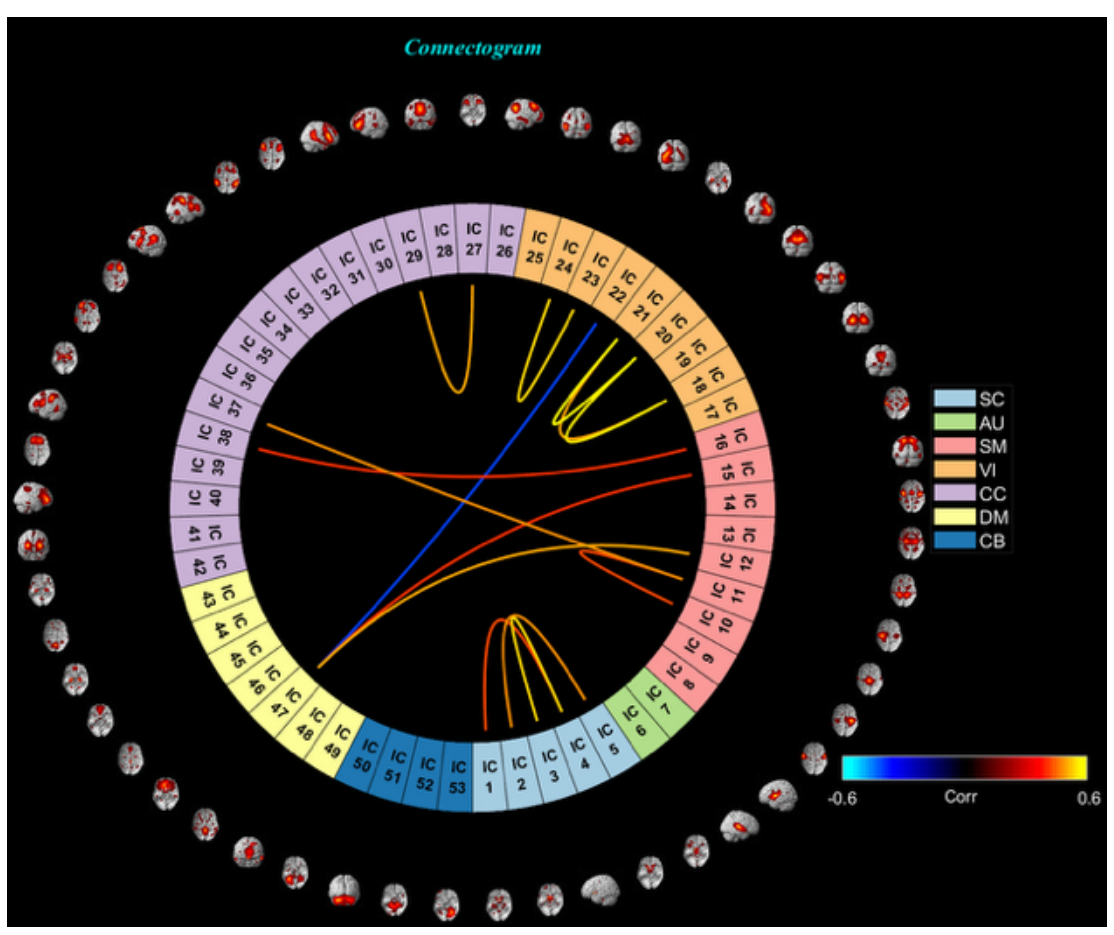

Figure 15: Component – 7: (a) FNC matrix and (c) connectogram reveals highly correlated regions are – hippocampus, postcentral gyrus, superior parietal lobule, anterior cingulate cortex, cuneus, occipital gyrus, fusiform gyrus. (b) represent mean GM maps for component 7 shows negative peak activation in CB and DM.
